# Management of People Who Inject Drugs With Serious Injection-Related Infections in an Outpatient Setting: A Scoping Review

**DOI:** 10.1093/ofid/ofae613

**Published:** 2024-10-10

**Authors:** Arunima Soma Dalai, Wayne Leung, Heather Johnson, Anthony D Bai

**Affiliations:** Division of Infectious Diseases, Department of Medicine, Queen's University, Kingston, Ontario, Canada; Division of Infectious Diseases, Department of Medicine, Western University, London, Ontario, Canada; Division of General Internal Medicine, Department of Medicine, Queen's University, Kingston, Ontario, Canada; Division of Infectious Diseases, Department of Medicine, Queen's University, Kingston, Ontario, Canada

**Keywords:** outpatient, people who inject drugs, serious injection-related infections, outpatient parenteral antimicrobial therapy

## Abstract

**Background:**

People who inject drugs (PWID) are at risk of severe injection-related infection (SIRI), which is challenging to manage. We conducted a scoping review to map the existing evidence on management of PWID with SIRI in an outpatient setting.

**Methods:**

We conducted a literature search in MEDLINE, Embase, Cochrane Central, and CINAHL from their inception until 6 December 2023. Studies were included if they focused on PWID with SIRI requiring ≥2 weeks of antibiotic therapy, with a proportion of management occurring outside hospitals. Studies were categorized inductively and described.

**Results:**

The review included 68 articles with the following themes. PWID generally prefer outpatient management if deemed safe and effective. Most studies support outpatient management, finding it to be as effective and safe as inpatient care, as well as less costly. Successful transition to outpatient management requires multidisciplinary discharge planning with careful consideration of patient-specific factors. Emerging evidence supports the effectiveness and safety of outpatient parenteral antibiotic therapy, long-acting lipoglycopeptides, and oral antibiotic therapy, each having unique advantages and disadvantages. Various specialized outpatient settings, such as skilled nursing facilities and residential treatment centers, are available for management of these infections. Finally, all patients are likely to benefit from adjunctive addiction care.

**Conclusions:**

Emerging evidence indicates that outpatient management is effective and safe for SIRI, which is preferred by most PWID. Key components of outpatient management include multidisciplinary discharge planning, appropriate antibiotic modality, suitable care settings, and adjunctive addiction care. These elements should be carefully tailored to patient needs and circumstances.

Over the past decade, hospitalizations for severe injection-related infections (SIRIs) have increased alongside the ongoing crisis of drug overdose deaths [1, 2]. SIRIs include bacteremia, endocarditis, deep abscesses, osteomyelitis, and septic arthritis, which frequently lead to lengthy hospital stays [3] with high readmission rates [4], patient-initiated discharges [3], and high postdischarge mortality [5]. Between 2007 and 2017 in the United States, the incidence of opioid use disorder–associated endocarditis increased by nearly 80% [6], with a projected 250 000 deaths by 2030 [7].

Outpatient parenteral antimicrobial therapy (OPAT) is commonly used for patients requiring prolonged intravenous (IV) antibiotics outside the hospital, typically administered via a peripherally inserted central catheter (PICC) [8]. However, people who inject drugs (PWID) and SIRI have historically been excluded from OPAT due to assumptions that these patients will inject illicit drugs into their PICCs or fail to adhere to prescribed antibiotic courses as outpatients [9–11]. Despite the growing impact of SIRIs, there remains no consensus on the best approach to manage this complex syndrome in an outpatient setting.

To date, no systematic or scoping review has explored the outpatient management of PWID with SIRI in terms of discharge planning, antibiotic delivery methods, settings, and adjunctive addiction treatment. Our aim is to identify and map the available evidence on this topic and highlight knowledge gaps for further research.

## METHODS

### Protocol and Registration

This scoping review was reported per the PRISMA-ScR guidelines (Preferred Reporting Items for Systematic Reviews and Meta-analyses Extension for Scoping Reviews) [12]. The study protocol was registered at the Center for Open Science (https://osf.io/e5c94/).

### Information Sources and Search

We conducted a literature search in OVID-MEDLINE, Embase, Cochrane Central Register of Controlled Trials, and CINAHL from inception until 6 December 2023. The search strategy was developed with a librarian using MeSH terms to capture concepts of PWID, bacterial infection syndromes, outpatient setting, and different antibiotic routes (Supplementary Tables 1–3). Reference lists from included studies were searched by hand to find any additional relevant studies.

### Eligibility Criteria

For this scoping review, we included peer-reviewed journal articles and conference abstracts in any language. Eligible study designs included case series, cohort studies, case-control studies, cross-sectional studies, quasi-experimental studies, mixed methods studies, qualitative studies, randomized controlled trials, systematic reviews, guidelines, position statements, and policy statements. We excluded commentaries, study protocols, and narrative reviews. Case reports were also excluded because they describe only 1 patient.

For the patient population and interventions, we included studies investigating PWID with SIRI (eg, bacteremia, endocarditis, deep abscesses, bone or joint infection) that required at least 2 weeks of antibiotic therapy based on the syndrome, in which a part of the treatment occurred outside of the hospital. PWID must be the only study population, the majority, or a separately analyzed subgroup for outcomes.

### Selection of Sources of Evidence

Citations were uploaded onto covidence citation management software. Two independent reviewers (A. S. D., W. L.) screened the title and abstracts to identify relevant studies for full-text reading. Disagreements on screening were resolved by a third reviewer (A. D. B.).

### Data-Charting Process and Data Items

Two independent reviewers read each full text and extracted data using a standardized form. Disagreements between reviewers were resolved by discussion to reach a consensus or by adjudication from a third reviewer if necessary.

Extracted variables were as follows: author names, publication year, journal name, study location, study period, study design, research question, definition of PWID population, sample size, infection syndrome, study intervention (antibiotic route and setting), substance use disorder (SUD) management, comparison group, outcomes, results, and conclusion on advantages and disadvantages associated with the study intervention.

### Critical Appraisal of Individual Sources

Critical appraisal of individual sources of evidence was not done due to the breadth of the scoping review, which comprised various study designs and research questions.

### Synthesis of Results

Studies were categorized by their research questions under the following themes: (1) patient and provider perspectives on outpatient management, (2) outpatient vs inpatient management, (3) discharge planning, (4) predictors of success or failure for outpatient management, (5) antibiotic delivery modalities, (6) type of outpatient setting, and (7) adjunctive SUD treatment. Given the heterogeneity across studies, a descriptive analysis of individual studies was done.

A critical interpretive synthesis approach [13] was used to develop a new theoretical conceptualization of a comprehensive and individualized approach to outpatient management of PWID with infections, as grounded in the studies in this review.

## RESULTS

**Figure 1.**
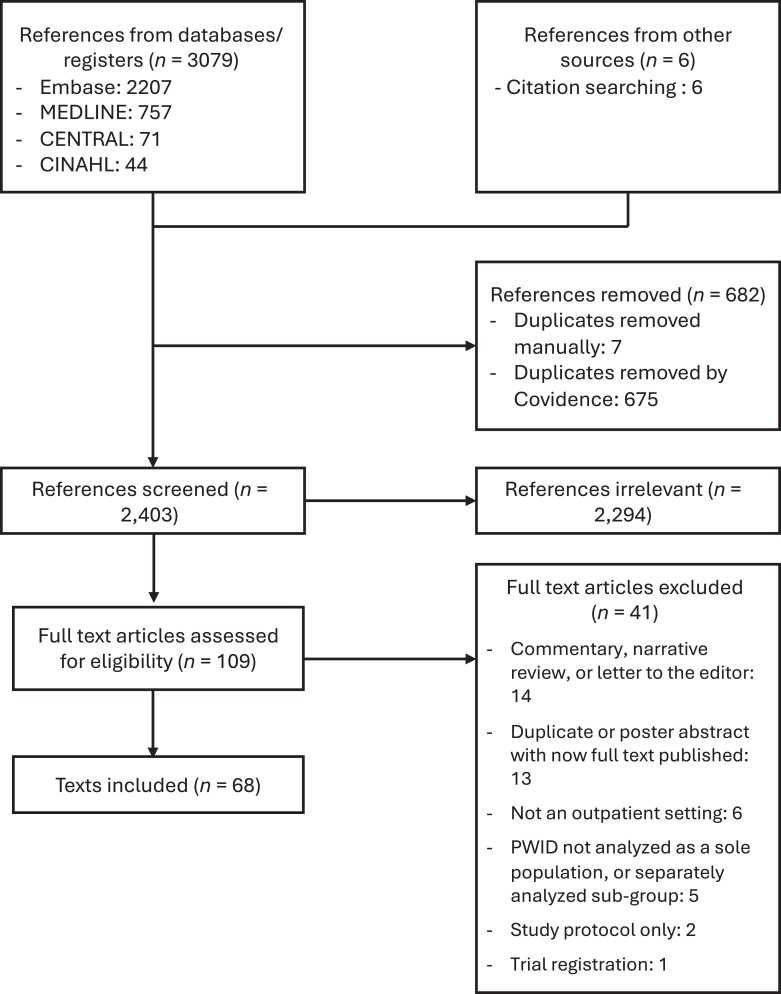
Flow diagram for inclusion of studies. PWID, people who inject drugs.

The literature search yielded 2403 unique articles (Figure 1). After screening and full-text reading, 68 studies were included in the scoping review [8, 14–80]. The 68 studies are described in Supplementary Table 4.

Of the 68 studies, there were 38 (56%) retrospective case series, 13 (19%) retrospective cohort studies, 4 (6%) quasi-experimental studies, 3 (4%) cross-sectional surveys, 2 qualitative studies, 1 decision analytic modeling study, 1 scientific statement, 1 prospective case series, 1 prospective cohort study, 1 societal guideline, 1 quality improvement and health care redesign study, 1 mixed methods study, and 1 preliminary result of a randomized trial. Sixty-three studies (91%) were conducted in the United States while the remainder were conducted in Australia, Singapore, Canada, or internationally. The median date of publication was 2019 (IQR, 2017–2021).

Studies typically explored multiple themes. Six (9%) studied perceptions of patients and care providers. Ten (15%) compared outpatient vs inpatient management of PWID. Twenty-one (31%) examined discharge planning. Ten (15%) analyzed predictive factors of success or failure in this population. Thirty-eight (56%) investigated different modalities of outpatient antibiotic modalities. Fourteen (21%) focused on types of outpatient setting for antibiotic treatment. Fourteen (20.3%) evaluated adjunctive SUD management.

### Patient and Provider Perspectives

Patients generally favored outpatient management when it was deemed safe and effective. In 1 study, 100% of patients believed that they received better care in a specialized outpatient setting as compared with the hospital [47]. In another study, patients reported that stigma, inadequate pain control, and decreased autonomy contributed to poor hospital experiences [35]. Patients expressed interest in OPAT if it was perceived to be equally as effective and if there were available resources and social support to support them in receiving OPAT [35, 47].

Provider perspectives regarding outpatient antibiotic treatment of PWID were complex and nuanced. Several barriers prevented outpatient treatment, such as fear of discharging patients to settings with unstable housing or lack of transportation [38, 55, 70], fear that patients would not adhere to antimicrobial regimens or would misuse PICC lines [38, 55, 70], and lack of awareness of existing resources [55, 70]. In addition, providers cited a lack of knowledge about evidence-based oral regimens [55], uncertainty about how responsibility for offering outpatient treatment is shared across care teams, and fear of medicolegal repercussions [38, 55]. Despite these barriers, 3 of 4 surveys of health care providers reported that 71.9% to 100% believed that OPAT or alternate models of outpatient antibiotic therapy should be considered for PWID, including a 2012 international survey of 64 OPAT centers [45, 47, 70]. However, a 2012 survey of 66 physicians in the United States found that only 12% of those surveyed would consider OPAT for PWID, as opposed to 95% in those without injection drug use [38]. A 2022 survey of 239 infectious disease clinicians in the United States found that those who believed that PWID were not eligible for OPAT had significantly less access to social workers, case management, and outpatient addiction services [70].

### Outpatient vs Inpatient Management

Ten studies compared complete inpatient treatment of PWID with prolonged IV antibiotics vs partial outpatient management that included OPAT, long-acting lipoglycopeptides, and partial oral antibiotic therapy [14, 34, 41, 51, 53, 67, 72, 73, 78, 79]. Overall, the studies were largely in favor of partial outpatient management, showing either no difference or even better outcomes in terms of mortality, clinical cure, cost, compliance, and readmission (Table 1).

**Table 1.** Inpatient vs Outpatient Management for Persons Who Use Drugs and PWID on Prolonged Antibiotics

Outcome	Evidence Supporting Outpatient or Inpatient Management
Mortality	**6 studies supporting outpatient management** In a retrospective cohort study of PWID with deep-seated infections that compared outpatient dalbavancin treatment with inpatient treatment, the outpatient group had a numerically lower all-cause 30-d mortality than the inpatient group (0/29 [0%] vs 2/20 [10%]). No statistical comparison was done [67]. In a decision analytic model and simulation study of 5 million patients with injection drug use–associated infective endocarditis that compared OPAT and partial oral antibiotic therapy with inpatient IV antibiotic treatment, the estimated attributable mortality rates were 4.89% in the OPAT group and 4.79% in the partial oral antibiotic therapy group, which were all lower than the 5.01% in the inpatient IV antibiotic treatment group [14] In a prospective quality improvement study of PWID with SIRI that compared 105 patients transitioned to oral antibiotics following patient-initiated discharge vs 61 patients who completed IV antibiotics in the hospital, there was no significant difference in 90-d mortality (*P* = .625). The number of deaths was not reported [51]. In a quasi-experimental study that compared the period before and after implementation of a drug recovery assistance and OPAT program, the 1-y all-cause mortality was numerically lower in the postintervention group (1/87 [1.1%] vs 4/51 [7.8%]), but this was not statistically significant (*P* = .06). In the propensity score–matched cohort, the mortality rate was 0 (0%) in the postintervention group and 2/31 (6.5%) in the preintervention group (*P* = .13) [41]. In a retrospective cohort study of PWID admitted with infective endocarditis, inpatient treatment was associated with a higher risk of 90-d mortality when compared with outpatient treatment with a hazard ratio of 3.39 (95% CI, 1.53–7.53) in a multivariable Cox regression model [73]. In a retrospective cohort study of PWID admitted with an invasive infection that compared patients who completed a full course of IV antibiotics as inpatients vs those who were prescribed oral antibiotics on patient-initiated discharge, mortality within 90 d after discharge was 2/83 (2%) in the partial oral antibiotic group and 7/143 (5%) in the IV antibiotic group (*P* = .489) [53].
Clinical cure	**3 studies supporting outpatient management** In a retrospective cohort study of PWID with deep-seated infections that compared outpatient dalbavancin treatment with inpatient treatment, the outpatient group had a numerically lower treatment failure than the inpatient group (2/29 [7%] vs 4/20 [20%]). No statistical comparison was done [67]. In a retrospective cohort study of PWID with complicated *Staphylococcus aureus* bacteremia that compared patients who had partial oral antibiotic therapy with patients who completed IV antibiotics as inpatients, microbiologic failure or death occurred for 9/69 (13%) in the partial oral antibiotic therapy group and 13/122 (11%) in the inpatient IV antibiotic group. This was not statistically significant after adjustment for other prognostic factors (adjusted odds ratio and *P* value not reported) [78]. In a retrospective cohort study of PWID with bone and joint infections that compared patients who received exclusively IV antibiotics in the hospital and patients who received partial oral antibiotics and could be discharged, the partial oral antibiotic group had a numerically lower clinical failure rate than the IV antibiotic group (13/74 [20%] vs 6/12 [50%]). No statistical comparison was done [79]. **1 inconclusive study** In a prospective quality improvement study of PWID with SIRI that compared 105 patients transitioned to oral antibiotics following patient-initiated discharge vs 61 patients who completed IV antibiotics in the hospital, the microbiologic failure rate was numerically higher in the oral antibiotic cohort (17/105 [16.2%] vs 6/61 [9.8%]), but this was not statistically significant (*P* = .434) [51]. **1 study supporting inpatient management** In a retrospective cohort study of PWID with orthopedic infections that compared patients who remained inpatient to complete antibiotic therapy vs those discharged to complete outpatient antibiotic treatment, the clinical cure rate at 6 months for the outpatient group was significantly lower than the inpatient group (11/17 [65%] vs 8/8 [100%], *P* = .0019). No difference in clinical cure rate was observed between the groups at 12 months, for which numbers were not reported [72].
Cost	**5 studies supporting outpatient management** In a decision analytic model and simulation study of 5 million patients with injection drug use–associated infective endocarditis that compared OPAT and partial oral antibiotic therapy with inpatient IV antibiotic treatment, the mean (95% credible interval) discounted cost was $412 150 ($331 540–$481 460) for OPAT and $413 920 ($333 220–$483 000) for partial oral antibiotic therapy, which were both less than $416 570 ($334 000–$482 780) for inpatient IV antibiotic treatment. OPAT was found to be cost saving [14]. In a prospective quality improvement study of PWID with SIRI that compared 105 patients transitioned to oral antibiotics following patient-initiated discharge vs 61 patients who completed IV antibiotics in the hospital, the mean (SD) direct inpatient cost was $28 415 ($30 183) for PWID treated with partial oral antibiotics vs $89 729 ($59 664) for those treated entirely as inpatients (*P* < .001) [51]. In a quasi-experimental study that compared the period before and after implementation of a drug recovery assistance and OPAT program, the total median (IQR) cost was significantly lower in the postintervention group ($39 220 [$23 300–$82 506] vs $27 592 [$18 509–$48 369], *P* = .007). In the propensity score–matched cohort, the total median (IQR) cost was significantly lower in the postintervention group ($68 748 [$34 485–$112 712] vs $33 231.88 [$24 170–$46 670], *P* < .0001) [41]. In a retrospective cohort study of PWID with orthopedic infections that compared patients who remained inpatient to complete antibiotic therapy vs those discharged to complete outpatient antibiotic treatment, the mean length of stay was 11.2 d in the outpatient group and 42.3 d in the inpatient group (*P* < .0001) [72]. In a quasi-experimental study that compared the period before and after implementation of an IV antibiotics and addiction team for PWID receiving IV antibiotics, which discharged patients with OPAT who were at low risk, the mean total direct cost per admission decreased from $38 716 to $26 014. No statistical comparison was made [34]. **1 inconclusive study** In a retrospective cohort study of PWID with deep-seated infections that compared outpatient dalbavancin treatment with inpatient treatment, the outpatient group had a numerically higher direct cost than inpatient treatment (median [IQR], $13 518 [$10 947–$19 387] vs $11 567 [$6348–$22 015]), but this was not statistically significant (*P* = .47) [67].
Compliance	**2 studies supporting outpatient management** In a retrospective cohort study of PWID with deep-seated infections that compared outpatient dalbavancin treatment with inpatient treatment, the outpatient group had a numerically higher treatment completion rate (19/29 [66%] vs 11/20 [55%]). No statistical comparison was done [67]. In a decision analytic model and simulation study of 5 million patients with injection drug use–associated infective endocarditis that compared OPAT and partial oral antibiotic therapy with inpatient IV antibiotic treatment, treatment completion was estimated to be 78.8% in OPAT group and 80.3% in partial oral antibiotic therapy group, which were both higher than the 77.6% in the inpatient group [14]. **1 study supporting inpatient management** In a retrospective cohort study of PWID with orthopedic infections that compared patients who remained inpatient to complete antibiotic therapy vs those discharged to complete outpatient antibiotic treatment, the outpatient group had a significantly lower treatment compliance rate than the inpatient group (11/23 [48%] vs 10/12 [83%], *P* = .0058) [72].
Complications	**1 study supporting outpatient management** In a retrospective cohort study of PWID admitted with infective endocarditis, inpatient treatment was associated with a significantly higher rate of new bloodstream infection vs OPAT, with a hazard ratio of 4.49 (95% CI, 2.30–8.76; *P* < .001) in a multivariable Cox regression model [73]. **1 inconclusive study** In a retrospective cohort study of PWID with orthopedic infections that compared patients who remained inpatient to complete antibiotic therapy vs those discharged to complete outpatient antibiotic treatment, the catheter complication rate for the outpatient group was numerically higher than the inpatient group (2/22 [9%] vs 0/12 [0%]), but this was not statistically significant (*P* = .1390) [72].
Readmission	**5 studies supporting outpatient management** In a retrospective cohort study of PWID with complicated *S aureus* bacteremia that compared patients who had partial oral antibiotic therapy vs patients who completed IV antibiotic as inpatients, readmission within 90 d occurred for 18/69 (26%) in the partial oral antibiotic therapy group and 38/122 (31%) in the inpatient IV antibiotic group (*P* = .02) [78]. In a prospective quality improvement study of PWID with SIRI that compared 105 patients transitioned to oral antibiotics following patient-initiated discharge vs 61 patients who completed IV antibiotics in the hospital, there was no significant difference in 90-d all-cause readmission rates in those treated with partial oral antibiotic therapy after patient-initiated discharge vs those who completed inpatient IV antibiotics (*P* = .739). Number of readmissions was reported in only the oral antibiotic cohort (26/105) [51]. In a quasi-experimental study that compared the period before and after implementation of a drug recovery assistance and OPAT program, the 90-d readmission rate was 21/87 (24%) in the postintervention group and 12/51 (24%) in the preintervention group (*P* = .80). In the propensity-matched cohort, the 90-d readmission rate was still similar (9/35 [26%] vs 6/31 [19.4%], *P* = .57) [41]. In a retrospective cohort study of PWID admitted with an invasive infection that compared patients who completed a full course of IV antibiotics as outpatients vs those who were prescribed oral antibiotics on patient-initiated discharge, the readmission rate was similar between the groups (45/143 [32%] vs 27/83 [33%]) with an adjusted hazard ratio of 0.99 (95% CI, .62–1.62) [53]. In a quasi-experimental study that compared the period before and after implementation of an IV antibiotics and addiction team for PWID receiving IV antibiotics, which discharged patients with OPAT who were at low risk, the 30-d readmission rate was numerically lower in the postintervention period than the preintervention period (18/99 [18%] vs 7/37 [19%]). No statistical comparison was made [34]. **2 studies supporting inpatient management** In a retrospective cohort study of PWID with deep-seated infections that compared outpatient dalbavancin treatment with inpatient treatment, the outpatient group had a numerically higher readmission rate within 90 d (6/29 [21%] vs 2/20 [10%]). No statistical comparison was done [67]. In a retrospective cohort study of PWID with orthopedic infections that compared patients who remained inpatient to complete antibiotic therapy vs those discharged to complete outpatient antibiotic treatment, the hospital readmission rate for the outpatient group was significantly higher than the inpatient group (1/12 [8%] vs 13/27 [48%], *P* = .0130) [72].
Loss to follow-up	**2 studies supporting outpatient management** In a retrospective cohort study of PWID with deep-seated infections that compared outpatient dalbavancin treatment with inpatient treatment, the outpatient group had a numerically lower rate of loss to follow-up (5/29 [17%] vs 7/20 [35%]). No statistical comparison was done [67]. In a retrospective cohort study of PWID with complicated *S aureus* bacteremia that compared patients who had partial oral antibiotic therapy with patients who completed IV antibiotic as inpatients, 5/69 (7%) in the partial oral antibiotic therapy group and 18/122 (15%) in the inpatient IV antibiotic group were lost to follow-up (*P* = .09) [78]. **2 studies supporting inpatient management** In a retrospective cohort study of PWID with orthopedic infections that compared patients who remained inpatient to complete antibiotic therapy vs those discharged to complete outpatient antibiotic treatment, loss to follow-up at 6 months occurred at a higher rate in the outpatient group than the inpatient group (12/29 [41%] vs 4/12 [33%]). No statistical comparison was done [72]. In a retrospective cohort study of PWID with bone and joint infections that compared patients who received exclusively IV antibiotics in the hospital vs patients who received partial oral antibiotics and could be discharged, the partial oral antibiotic group had a numerically higher rate of loss to follow-up than the IV antibiotic group (6/86 [7%] vs 0/12 [0%]). No statistical comparison was done [79].

Abbreviations: IV, intravenous; OPAT, outpatient antimicrobial therapy; PWID, people who inject drugs; SIRI, serious injection-related infection.

### Discharge Planning

Twenty-one studies discussed discharge planning for this complex population (Table 2). These studies employed diverse approaches, including multidisciplinary “tumor board”–style discharge panels [15] or simply prescribing oral antibiotics for patient-initiated discharges [51, 53, 78, 79]. Four studies used a structured model such as OPTIONS-DC [33, 69] or a 9-point risk assessment [34, 65] to identify patients suitable for discharge, while others adopted various approaches based on different patient factors.

**Table 2.** Factors that May Favor One Antibiotic Modality Over Another for Selection of Outpatient Treatment for Patients

OPAT	Long-acting Lipoglycopeptides	Oral Antibiotics	Not Eligible for Discharge
Safe home environment/social supports and transportation [21, 28, 33, 34, 44, 50, 58, 62, 63, 65, 69, 71] Engagement with addiction team, including uptake of MOUD [15, 28, 33, 34, 40, 62, 65, 69, 71] Ability to contact patient/follow-up [15, 33, 40, 44, 50, 58, 62, 69, 71] Engagement with care team [33, 40, 50, 58, 69] PICC safety/overdose prevention strategy in place [33, 40, 44, 63, 65, 69] Patient preference [21, 33, 50, 61, 69] No equivalent oral alternative [58] Capacity to understand risks/benefits of treatment options [50]	Identified source of infection [67] Inability to care for PICC [67] Inability to be discharged to a post–acute care facility [67]	Source control achieved [79] Patient-initiated discharge [51, 53, 78, 79] After ID evaluation and after 2–3 weeks of IV treatment [49, 66] Oral treatment is appropriate according to syndrome and culture results [49, 58] Patient agreeable and able to comply with follow-up [49, 58] No concerns regarding aggressive behavior or ongoing illicit drug or stimulant use that may impair compliance [58]	Active or severe stimulant, benzodiazepine, or alcohol use disorder [28, 34, 50, 58, 65] Dual/poorly controlled psychiatric diagnoses [28, 34, 63] Lack of social supports or stable housing [21, 50] No access to reliable communication [50] Not willing to engage with care team [50] Current incarceration [50] Cardiac surgery during this admission [50] History of poor compliance [28]

Abbreviations: ID, infectious disease; IV, intravenous; MOUD, medications for opioid use disorder; OPAT, outpatient antimicrobial therapy; PICC, peripherally inserted central catheter.

Regarding initiation of discharge planning, most studies allowed any member of the treating team to commence the process [33, 44, 58, 61, 69]. The next-most common processes were conducting formalized multidisciplinary reviews [40, 50, 62, 65] or being flagged by the infectious disease consultant for discharge options [28, 49, 51, 71]. The remainder used flagging systems by the addiction team [34, 71], antimicrobial stewardship team [67], or OPAT nursing team [50]. One guideline recommended integrating screening tools into the hospital admission workflow [21].

Once initiated, discharge planning involved infectious disease consultations in a majority of studies [21, 28, 33, 40, 49–51, 61, 62, 65, 67, 69, 71], followed by addictions medicine [15, 21, 28, 33, 40, 51, 58, 61, 62, 64, 65, 69, 71], case management [33, 40, 50, 51, 61, 62, 64, 67, 69], health coaches and peer recovery specialists [33, 50, 51, 64, 69], the OPAT/IV antibiotic team [33, 40, 50, 61, 62, 65, 69], psychiatry [15, 21, 28, 40, 50, 62], antimicrobial stewardship [49, 67], social workers [50, 65], the inpatient nursing team [50, 61], clinical liaisons between hospital and home health [50, 67], pharmacists [49, 61], and the risk management team [62].

Table 2 summarizes factors considered during discharge planning for OPAT, long-acting lipoglycopeptides, oral antibiotics, and inpatient care. The most commonly cited factors influencing OPAT discharge were the availability of social supports (including housing), engagement with the addiction team, uptake of medications for opioid use disorder (MOUD), and the ability to adhere to follow-up appointments.

### Predictors of Success or Failure for Outpatient Management

Ten studies commented on predictors of success and failure in outpatient management, defined as antibiotic completion, clinical cure, and readmissions [27, 29, 31, 41, 42, 51–53, 75, 79]. Three studies found that participation in outpatient MOUD was predictive of success [42, 51, 53], although 1 study suggested that it did not affect antibiotic completion [75]. Factors predictive of failure were stimulant use, polysubstance use disorder, homelessness [75], and patient-initiated discharges [27, 29, 31, 41, 52, 53, 75, 79], while surgical source control, engagement with a multidisciplinary team, discharge to medical respite, and infectious disease or surgical follow-up were predictive of success [51, 53, 79]. In the special circumstance when discharge was mediated by release to medical respite or a detoxification facility, homelessness was associated with success [41, 79].

### Antibiotic Delivery Modalities

A total of 38 studies examined outcomes in PWID treated as outpatients using various antibiotic modalities: 16 focused on OPAT, 18 on long-acting lipoglycopeptides, and 4 on partial oral antibiotic therapy. Table 3 summarizes the advantages and disadvantages for each approach.

**Table 3.** Outcomes, Advantages, and Disadvantages for Outpatient Antibiotic Modalities

Modality	Outcomes	Advantages	Disadvantages
OPAT	Clinical cure ranges from 56% to 100% [14, 23, 31, 44, 60–62, 71, 77] Treatment completion ranges from 66% to 100% [14, 20, 31, 41, 42, 44, 58, 61, 67, 72, 77] No statistical difference in clinical cure between IDU and non-IDU [31, 71, 77] PICC tampering/catheter complications range from 0% to 20% [23, 44, 58, 61, 62, 73, 77] Readmission rate ranges from 11% to 50% [15, 23, 41, 44, 58, 60, 72, 77, 80] Secondary bloodstream infection ranges from 4.2% to 19.5% [23, 73] Cost savings: $33 000 per patient [61]	Cost savings and/or decreased length of stay [14, 15, 31, 41, 44, 61, 62] Ability to link to addiction care [15, 40, 41, 71] Patient preference [35, 47, 62] Similarity in treatment outcomes to non-IDUs with OPAT [31, 71, 77] Increased life expectancy and prevention of fatal overdose [14] Allows for safety and compliance check [61]	Noncompliance/loss to follow-up [15, 20, 22, 31, 60, 63, 72] Readmissions and/or increased health care utilization for those noncompliant with treatment [15, 20, 58, 80] Potentially high rates of catheter blockage, damage, accidental removal/dislodgment [31] Line-associated infections [58, 73] Difficulty obtaining IV access [72] Antibiotics that require frequent dosing may be challenging to administer in OPAT [61] Transport to/from infusion center [61] Requirement for housing, social support, and insurance [58]
Long-acting lipoglycopeptides	Clinical cure ranges from 44.4% to 87.5% [16–18, 25, 26, 52, 56, 57, 68, 76] Treatment completion increased from 53% to 66% [67] Overall treatment completion 53% [26] 30-d readmission ranges from 5.8% to 19% [26, 56, 66, 74] Adverse effects ranges from 0% to 66% [16, 26, 43, 57, 66, 68] Reduction in average length of stay by 20–22.4 d per patient [54, 56]	Cost savings/decreased length of stay [52, 54, 56, 59, 68, 74] Ease of administration in patients with concerns about PICC lines, adherence, or inability to place in skilled nursing facility [16–18, 56] Prevention of readmission [59]	Nursing coordination time, which is estimated to average 118 min per patient [32] Challenges in IV access for each administration dose [32] Side effects requiring transition to oral therapy [25, 66] Loss to follow-up [43] Theoretical risk of inducible resistance if drug concentration decreases below minimum inhibitory concentration prior to infection clearance [17, 52, 56]
Oral antibiotic therapy	Clinical cure rate ranges from 79% to 87% [78, 79] Readmission rate ranges from 26% to 33% [53, 78] In 72% of patient-initiated discharges, patients still filled antibiotic prescription [20]	Can be offered to those who decline IV antibiotics or where there is a lack of safe/stable housing, health insurance, or access to outpatient follow-up [53, 78, 79] Improved clinical outcomes and decreased readmission rates vs patient-initiated discharge without oral antibiotics [53, 78, 79] Avoidance of complexities of OPAT (follow-up, PICC insertion, catheter complication) [72, 78] Cost savings/decreased length of stay [49]	Outpatient support to ensure that patients initiate and tolerate antibiotics may not be available at all institutions [53, 78] Oral regimens are not always available or tolerated [53, 56, 58] Complicated regimens are difficult to adhere to in populations with limited health literacy or cognitive impairment [78] Consistent adherence may not be obtained [72, 78] Higher loss to follow-up [78] Cost of filling outpatient prescription may be prohibitive [53]

Abbreviations: IDU, injection drug use; OPAT, outpatient parenteral antimicrobial therapy; PICC, peripherally inserted central catheter.

### Type of Outpatient Setting

Fourteen studies discussed special accommodation settings for outpatient antibiotic delivery, such as medical respite, skilled nursing facilities, residential treatment centers, detoxification facilities, home, “home-like settings,” and rural settings [19, 20, 22, 23, 28, 30, 36, 41, 46–49, 72, 75]. Definitions for these settings were inconsistent, and many facilities imposed restrictions, such as acceptance criteria for PWID or homeless patients, limitations on administering IV antibiotics, limited or lack of addictions training, stigma, curfews, visitor restrictions, restrictions on mobile device and internet use, random drug screening, and inconsistent physician staffing [23, 36, 48]. Many patients declined these settings due to these imposed restrictions [36]. However, these settings can offer benefits such as a stabilizing environment, multidisciplinary teams, access to addiction services and counseling, wound care, and shelter for unhoused individuals [20, 22, 23, 41, 47, 72, 75]. Three studies noted high rates of IV antibiotic completion at home in specially selected populations [30, 46, 72].

Other than accommodation settings, 3 studies described unique challenges in rural locations. Rural locations were noted to have limited access to MOUD/addictions specialists and traditional OPAT infrastructure [19, 28, 49].

### Adjunctive SUD Treatment

Fourteen studies focused on adjunctive SUD treatment, including themes of MOUD, peer or patient navigators, and psychiatry referrals [9, 14, 15, 21, 36, 39–42, 61, 62, 64, 71, 75]; all except 1 study were retrospective. Outpatient SUD treatment may increase patient engagement with addiction care [14, 15] and improve treatment acceptability [61]. Literature on whether SUD treatment improves outcomes for PWID with SIRI is conflicting: some studies showed improvement in hospital readmission rates, OPAT completion, and reduction in infection recurrence [42, 62, 64], while others found no significant differences [41, 75]. One study even reported an increase in readmissions with SUD treatment [15]. Preliminary findings from a randomized controlled trial in which 37 patients were randomized to buprenorphine-OPAT suggested fewer serious adverse events and reduced length of stay when compared with inpatient management, although outcomes regarding infection are still pending [37, 39]. The American Heart Association’s 2022 scientific statement on management of infective endocarditis in PWID recommended SUD treatment in all cases [21]. Few studies discussed how many patients remained in remission during the follow-up period, with reported relapse rates ranging from 11% to 15% in 2 studies [62, 71].

### Interpretive Synthesis

**Figure 2.**
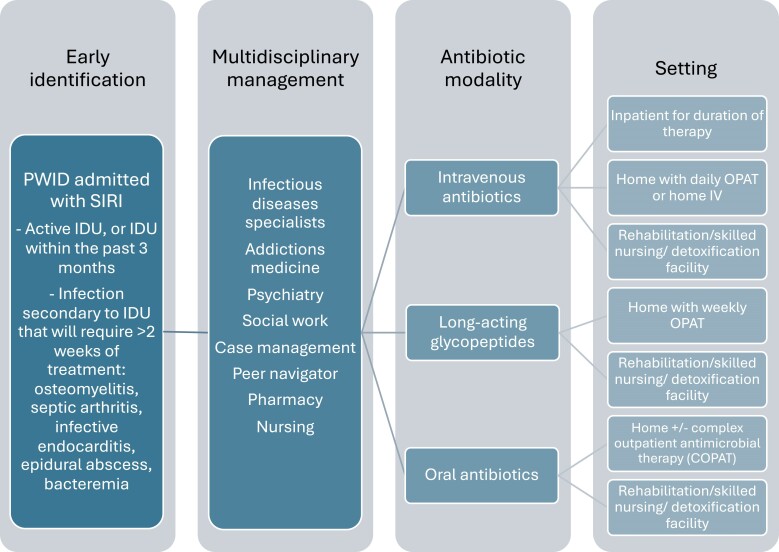
Important components of outpatient management of PWID with SIRI. IDU, injection drug use; OPAT, outpatient parenteral antibiotic therapy; PWID, people who inject drugs; SIRI, serious injection-related infection.

Figure 2 summarizes the care of PWID hospitalized with SIRI based on the aforementioned results. Important components of management were early identification of PWID with SIRI during the hospital stay, multidisciplinary discharge planning with careful patient selection, and tailoring antibiotic modalities as well as settings to the patients.

## DISCUSSION

Our scoping review describes emerging evidence that supports effective and safe care for PWID with SIRI in outpatient settings. We identified important components of outpatient care, such as multidisciplinary discharge planning, antibiotic modalities, setting, and SUD treatment, which should be tailored to the patient.

In a 2018 systematic review on OPAT in PWID, Suzuki et al [10] found that 72% to 100% of PWID successfully completed OPAT among 10 studies, which was comparable to reported OPAT outcomes among patients without injection drug use. In contrast, our scoping review was broader and examined other components of outpatient care as well as other antibiotic modalities such as partial oral antibiotic therapy. In addition, our review included studies of OPAT in the 6 years since the systematic review by Suzuki et al. In a 2020 narrative review, Hurley et al [81] reinforced the conclusions of Suzuki et al, stating that “there is no convincing data that inpatient stays are superior to OPAT in people with substance use disorders.” The authors additionally outlined outpatient treatment approaches, such as long-acting IV antibiotics, oral therapy, residential treatment settings, and infusion centers. However, this review was not systematic and did not examine qualitative aspects of patient attitudes toward outpatient therapy.

Strengths of our scoping review include its breadth, which encompassed multiple aspects of outpatient antibiotic delivery: OPAT, partial oral treatment, long-acting lipoglycopeptides, discharge planning, different outpatient settings, adjunctive SUD treatment, and patient/provider perspectives. The study also was rigorous and conducted per the PRISMA-ScR guidelines [12]. Under the guidance of a librarian, we conducted a comprehensive literature search in multiple databases with no language restrictions. Two independent reviewers screened studies, read the full text, and collected the data. A critical interpretive synthesis approach [13] was used to develop a new theoretical conceptualization of a comprehensive and individualized approach to outpatient management of PWID with SIRI based on the key themes identified.

There are several limitations that merit mentioning. First, as a scoping review that included various study designs, we did not formally assess the study quality, as it would be outside the scope of the review. However, in general, most studies were retrospective observational studies, often without appropriate comparison groups, which would be considered poor quality evidence. In addition, the outcomes likely are not comparable among studies due to variability in patient population, type of infectious syndrome, and rate of loss to follow-up. Second, our results are largely American-centric, as 91% of the studies were conducted in the United States. Globally, it is estimated that there are currently 15.6 million PWID aged 15 to 64 years [82]. Thus, the studies to date on mostly PWID in the United States represent a small proportion of this population.

Our review suggests that increased support for this population in outpatient settings, such as multidisciplinary teams, availability of SUD treatment, patient-centered rehabilitation, or detoxification facilities, can improve patient outcomes and reduce costs through shorter hospital stays, fewer readmissions, and fewer complications. Providers should develop patient-centered approaches to infection treatment and prioritize SUD treatment; they should also assemble multidisciplinary teams to assess psychiatric comorbidities, housing, social support, and possible transition to outpatient management. Hospitals should increase support for PWID discharged with PICC lines, including nursing support and linkage to outpatient addiction services and social work/case management, to increase provider confidence in discharging these complex patients. Health policy makers should consider increasing the availability for MOUD (including ease of licensing and prescription) and community infrastructure for detoxification/rehabilitation beds, especially for unhoused patients to increase adherence [41, 79].

Providers seem to already be moving in the direction suggested by our review, with most surveys showing that OPAT or alternate models of outpatient therapy should be considered in this population. While PICC tampering is a common concern, PICC subversion rates generally were low between 0% and 6% in most studies [44, 58, 61, 62, 71]. Inpatient management does not prevent PICC tampering or PICC line–associated bloodstream infection based on the existing evidence. In a retrospective study of PWID receiving parenteral antibiotic therapy for infective endocarditis, ongoing inpatient injection drug use was documented by the physician in 46% of cases, and new bloodstream infection occurred more frequently for patients who completed treatment in the hospital than patients who received OPAT [73]. Increased support from social work, case management, and outpatient addiction services may help enhance confidence in outpatient treatment options [70].

Our scoping review identified several knowledge gaps. There are few published studies on partial oral antibiotic therapy for PWID with SIRI. Future research should focus on prospective studies with appropriate comparison groups and adequate follow-up, given that many of these retrospective studies had significant proportions of their study patients lost to follow-up. This could be improved with linkage to case managers/social workers in an outpatient setting. Future studies may explore other approaches that have not been well studied, such as use of peer navigators or free cell phones. Research should also prioritize patient-important outcomes, such as clinical cure, infection recurrence, adverse events, functional status, and quality of life, rather than institution-centered outcomes such as cost savings or length of stay. Including patient voices and advocates in the study design could orient future study outcomes to what matters most for patients and potentially improve this population's trust in health care institutions.

## Supplementary Data

Supplementary materials are available at *Open Forum Infectious Diseases* online. Consisting of data provided by the authors to benefit the reader, the posted materials are not copyedited and are the sole responsibility of the authors, so questions or comments should be addressed to the corresponding author.

## Supplementary Material

ofae613_Supplementary_Data

## Notes

***Acknowledgments*.** We thank Sarah Wickett, the health informatics librarian at Queen's University Bracken Health Sciences Library, for her help with the development of the literature search strategy for this study.

***Author contributions.*** A. S. D. and A. D. B. conceived and designed the study. A. S. D., A. D. B., and W. L. performed the data collection. A. S. D. performed the data analysis. All authors contributed to the interpretation of results. A. S. D. wrote the first draft of the manuscript. All authors contributed to the revision of manuscript drafts.

***Data availability.*** Data are available in the supplement materials.

## References

[1] Wurcel AG, DeSimone DC, Marks L, Baddour LM, Sendi P. Which trial do we need? Long-acting glycopeptides versus oral antibiotics for infective endocarditis in patients with substance use disorder. Clin Microbiol Infect 2023; 29:952–4. 37044275 10.1016/j.cmi.2023.04.005

[2] Shiels MS, Freedman ND, Thomas D, Berrington de Gonzalez A. Trends in US drug overdose deaths in non-Hispanic Black, Hispanic, and non-Hispanic White persons, 2000–2015. Ann Intern Med 2018; 168:453–5. 29204603 10.7326/M17-1812 PMC6309971

[3] Schranz AJ, Wu L-T, Wohl D, Rosen DL. Readmission after discharge against medical advice for persons with opioid-associated infective endocarditis. Circulation 2019; 140(Suppl 1):A11954.

[4] Leahey PA, LaSalvia MT, Rosenthal ES, Karchmer AW, Rowley CF. High morbidity and mortality among patients with sentinel admission for injection drug use–related infective endocarditis. Open Forum Infect Dis 2019; 6:ofz089. 30949535 10.1093/ofid/ofz089 PMC6441563

[5] Straw S, Baig MW, Gillott R, et al Long-term outcomes are poor in intravenous drug users following infective endocarditis, even after surgery. Clin Infect Dis 2020; 71:564–71. 31504326 10.1093/cid/ciz869

[6] Wong CY, Zhu W, Aurigemma GP, et al Infective endocarditis among persons aged 18–64 years living with human immunodeficiency virus, hepatitis C infection, or opioid use disorder, United States, 2007–2017. Clin Infect Dis 2021; 72:1767–81. 32270861 10.1093/cid/ciaa372

[7] Barocas JA, Eftekhari Yazdi G, Savinkina A, et al Long-term infective endocarditis mortality associated with injection opioid use in the United States: a modeling study. Clin Infect Dis 2021; 73:e3661–9. 32901815 10.1093/cid/ciaa1346 PMC8662770

[8] Norris AH, Shrestha NK, Allison GM, et al 2018 Infectious Diseases Society of America clinical practice guideline for the management of outpatient parenteral antimicrobial therapy. Clin Infect Dis 2019; 68:e1–35. 10.1093/cid/ciy745 30423035

[9] Fanucchi LC, Lofwall MR, Nuzzo PA, Walsh SL. In-hospital illicit drug use, substance use disorders, and acceptance of residential treatment in a prospective pilot needs assessment of hospitalized adults with severe infections from injecting drugs. J Subst Abuse Treat 2018; 92:64–9. 30032946 10.1016/j.jsat.2018.06.011

[10] Suzuki J, Johnson J, Montgomery M, Hayden M, Price C. Outpatient parenteral antimicrobial therapy among people who inject drugs: a review of the literature. Open Forum Infect Dis 2018; 5:ofy194. 30211247 10.1093/ofid/ofy194 PMC6127783

[11] Tice AD, Rehm SJ, Dalovisio JR, et al Practice guidelines for outpatient parenteral antimicrobial therapy: IDSA guidelines. Clin Infect Dis 2004; 38:1651–71. 15227610 10.1086/420939

[12] Tricco AC, Lillie E, Zarin W, et al PRISMA Extension for Scoping Reviews (PRISMA-ScR): checklist and explanation. Ann Intern Med 2018; 169:467–73. 30178033 10.7326/M18-0850

[13] Dixon-Woods M, Cavers D, Agarwal S, et al Conducting a critical interpretive synthesis of the literature on access to healthcare by vulnerable groups. BMC Med Res Methodol 2006; 6:35. 16872487 10.1186/1471-2288-6-35 PMC1559637

[14] Adams JW, Savinkina A, Hudspeth JC, et al Simulated cost-effectiveness and long-term clinical outcomes of addiction care and antibiotic therapy strategies for patients with injection drug use–associated infective endocarditis. JAMA Netw Open 2022; 5:e220541. 35226078 10.1001/jamanetworkopen.2022.0541 PMC8886538

[15] Agrawal A, Fournier O, Landsman HS, et al (27) There's no place like home: a multidisciplinary approach to holistically manage addiction and intravenous drug use related infections requiring intravenous antibiotics. J Acad Consult Liaison Psychiatry 2022; 63(Suppl 2):S125–6.

[16] Ahiskali A, Rhodes H. Oritavancin for the treatment of complicated gram-positive infection in persons who inject drugs. BMC Pharmacol Toxicol 2020; 21:73. 33115540 10.1186/s40360-020-00452-z PMC7594421

[17] Ajaka L, Heil E, Schmalzle S. Dalbavancin in the treatment of bacteremia and endocarditis in people with barriers to standard care. Antibiotics (Basel) 2020; 9:700. 33076275 10.3390/antibiotics9100700 PMC7602462

[18] Alosaimy S, Pearson J, Veve M, et al Real-world experience with dalbavancin for complicated gram-positive infections: a multicenter evaluation. Open Forum Infect Dis 2019; 6(Suppl 2):S118–9.

[19] AlSalman A, Worby CP, Considine E, Zijoo R, Kershaw C. Dalbavancin utilization in rural healthcare setting: a single center three years’ experience. Open Forum Infect Dis 2020; 7(Suppl 1):S362.

[20] Ashraf B, Hoff E, Brown LS, et al Health care utilization patterns for patients with a history of substance use requiring OPAT. Open Forum Infect Dis 2021; 8:ofab540. 35559131 10.1093/ofid/ofab540 PMC9088504

[21] Baddour LM, Weimer MB, Wurcel AG, et al Management of infective endocarditis in people who inject drugs: a scientific statement from the American Heart Association. Circulation 2022; 146:e187–201. 36043414 10.1161/CIR.0000000000001090

[22] Beieler A, Magaret A, Zhou Y, Schleyer A, Wald A, Dhanireddy S. Outpatient parenteral antimicrobial therapy in vulnerable populations—people who inject drugs and the homeless. J Hosp Med 2019; 14:105–9. 30785418 10.12788/jhm.3138 PMC6996559

[23] Beieler AM, Dellit TH, Chan JD, et al Successful implementation of outpatient parenteral antimicrobial therapy at a medical respite facility for homeless patients. J Hosp Med 2016; 11:531–5. 27120700 10.1002/jhm.2597

[24] Bird C, Collins R, Mang N, Nijhawan AE, Bhavan K. Patients with substance use disorder discharged from safety net hospital to skilled nursing facility for OPAT: baseline characteristics and clinical outcomes. Open Forum Infect Dis 2019; 6(Suppl 2):S340.

[25] Bork JT, Heil EL, Berry S, et al Dalbavancin use in vulnerable patients receiving outpatient parenteral antibiotic therapy for invasive gram-positive infections. Infect Dis Ther 2019; 8:171–84. 31054088 10.1007/s40121-019-0247-0 PMC6522607

[26] Bryson-Cahn C, Beieler AM, Chan JD, Harrington RD, Dhanireddy S. Dalbavancin as secondary therapy for serious *Staphylococcus aureus* infections in a vulnerable patient population. Open Forum Infect Dis 2019; 6:ofz028. 30838225 10.1093/ofid/ofz028 PMC6388764

[27] Buehrle D, Shields RK, Shah N, Shoff C, Sheridan K. Risk factors associated with outpatient parenteral antibiotic therapy (OPAT) program failure among intravenous drug users (IVDUS). Open Forum Infect Dis 2016; 3(Suppl 1):1333. 10.1093/ofid/ofx102 PMC5493937 28680904

[28] Camsari UM, Libertin CR. Small-town America's despair: infected substance users needing outpatient parenteral therapy and risk stratification. Cureus 2017; 9:e1579. 29057191 10.7759/cureus.1579 PMC5647128

[29] Narayanan S, Ching PR, Traver EC, George N, Amoroso A, Kottilil S. Predictors of nonadherence among patients with infectious complications of substance use who are discharged on parenteral antimicrobial therapy. Open Forum Infect Dis 2023; 10:ofac633. 36686627 10.1093/ofid/ofac633 PMC9845962

[30] D’Couto HT, Robbins GK, Ard KL, Wakeman SE, Alves J, Nelson SB. Outcomes according to discharge location for persons who inject drugs receiving outpatient parenteral antimicrobial therapy. Open Forum Infect Dis 2018; 5:ofy056. 29766017 10.1093/ofid/ofy056 PMC5941140

[31] Dobson PM, Loewenthal MR, Schneider K, Lai K. Comparing injecting drug users with others receiving outpatient parenteral antibiotic therapy. Open Forum Infect Dis 2017; 4:ofx183. 29026870 10.1093/ofid/ofx183 PMC5632303

[32] Douglass A, Mayer H, Young K, et al The hidden cost of dalbavancin: oPAT-RN time spent on coordination for patients with substance use disorder. Open Forum Infect Dis 2021; 8(Suppl 1):S416–7.

[33] Douglass AH, Mayer H, Young K, et al A review of antibiotic outcomes data utilizing the multidisciplinary OPTIONS-DC conference for PWUD. Open Forum Infect Dis 2022; 9(Suppl 2):S681–2.

[34] Eaton E, Mathews R, Lane PS, et al A 9-point risk assessment for patients who inject drugs requiring intravenous antibiotics may allow health systems to focus inpatient resources on those at greatest risk of ongoing drug use. Open Forum Infect Dis 2018; 5(Suppl 1):S43–4.

[35] Eckland A, Kohut M, Stoddard H, et al “I know my body better than anyone else”: a qualitative study of perspectives of people with lived experience on antimicrobial treatment decisions for injection drug use-associated infections. Ther Adv Infect Dis 2023; 10:20499361231197065. 37693858 10.1177/20499361231197065 PMC10492466

[36] Englander H, Wilson T, Collins D, et al Lessons learned from the implementation of a medically enhanced residential treatment (MERT) model integrating intravenous antibiotics and residential addiction treatment. Subst Abuse 2018; 39:225–32. 10.1080/08897077.2018.1452326 PMC6519053 29595367

[37] Fanucchi LC, Murphy SM, Surratt H, et al Design and protocol of the buprenorphine plus outpatient parenteral antimicrobial therapy (B-OPAT) study: a randomized clinical trial of integrated outpatient treatment of opioid use disorder and severe, injection-related infections. Ther Adv Infect Dis 2022; 9:20499361221108005. 35847566 10.1177/20499361221108005 PMC9277431

[38] Fanucchi L, Leedy N, Li J, Thornton AC. Perceptions and practices of physicians regarding outpatient parenteral antibiotic therapy in persons who inject drugs. J Hosp Med 2016; 11:581–2. 27043146 10.1002/jhm.2582

[39] Fanucchi LC, Walsh SL, Thornton AC, Nuzzo PA, Lofwall MR. Outpatient parenteral antimicrobial therapy plus buprenorphine for opioid use disorder and severe injection-related infections. Clin Infect Dis 2020; 70:1226–9. 31342057 10.1093/cid/ciz654 PMC7931831

[40] Fanucchi LC, Walsh SL, Thornton AC, Lofwall MR. Integrated outpatient treatment of opioid use disorder and injection-related infections: a description of a new care model. Prev Med 2019; 128:105760. 31251946 10.1016/j.ypmed.2019.105760

[41] Gelman SS, Stenehjem E, Foster RA, Tinker N, Grisel N, Webb BJ. A novel program to provide drug recovery assistance and outpatient parenteral antibiotic therapy in people who inject drugs. Open Forum Infect Dis 2022; 9:ofab629. 35106314 10.1093/ofid/ofab629 PMC8801220

[42] Greco CS, Sobhanie MM, Coe KE, Hebert C, Williams M. The effect of medication-assisted treatment on completion rates of outpatient parenteral antibiotic therapy. Open Forum Infect Dis 2021; 8(Suppl 1):S403.

[43] Heil E, Martinelli A, Oliver W, Claeys K. Emergency department resource utilization after implementation of a dalbavancin pathway for skin and soft-tissue infections. Open Forum Infect Dis 2018; 5(Suppl 1):S703.

[44] Ho J, Archuleta S, Sulaiman Z, Fisher D. Safe and successful treatment of intravenous drug users with a peripherally inserted central catheter in an outpatient parenteral antibiotic treatment service. J Antimicrob Chemother 2010; 65:2641–4. 20864497 10.1093/jac/dkq355

[45] Ho J, Archuleta S, Tice A, Fisher D. International approaches to treating intravenous drug users in outpatient parenteral antibiotic services. Infect Dis Clin Pract 2012; 20:192–5.

[46] Hoff E, Ashraf B, Smartt J, Marambage K, Bhavan K. Empowering patients with addiction to self-administer parenteral antibiotics at home: a pilot project. Open Forum Infect Dis 2021; 8(Suppl 1):S409–10.

[47] Jafari S, Joe R, Elliot D, Nagji A, Hayden S, Marsh DC. A community care model of intravenous antibiotic therapy for injection drug users with deep tissue infection for “reduce leaving against medical advice.” Int J Ment Health Addict 2015; 13:49–58. 10.1007/s11469-014-9511-4 PMC4320270 25685126

[48] Jewell C, Weaver M, Sgroi C, Anderson K, Sayeed Z. Residential addiction treatment for injection drug users requiring intravenous antibiotics: a cost-reduction strategy. J Addict Med 2013; 7:271–6. 23648642 10.1097/ADM.0b013e318294b1eb

[49] Juskowich JJ, Ward A, Spigelmyer AE, et al Complex outpatient antimicrobial therapy (COpAT) program at a rural academic medical center: evaluation of first 100 patients. Open Forum Infect Dis 2022; 9(Suppl 2):S418–9.

[50] Kershaw C, Lurie JD, Brackett C, et al Improving care for individuals with serious infections who inject drugs. Ther Adv Infect Dis 2022; 9:20499361221142476. 36600726 10.1177/20499361221142476 PMC9806364

[51] Lewis S, Liang SY, Schwarz ES, et al Patients with serious injection drug use-related infections who experience patient-directed discharges on oral antibiotics have high rates of antibiotic adherence but require multidisciplinary outpatient support for retention in care. Open Forum Infect Dis 2022; 9:ofab633. 35106316 10.1093/ofid/ofab633 PMC8801224

[52] Lueking R, Wei W, Mang NS, Ortwine JK, Meisner J. Evaluation of dalbavancin use on clinical outcomes, cost-savings, and adherence at a large safety net hospital. Microbiol Spectr 2023; 11:e0238522. 36537818 10.1128/spectrum.02385-22 PMC9927367

[53] Marks LR, Liang SY, Muthulingam D, et al Evaluation of partial oral antibiotic treatment for persons who inject drugs and are hospitalized with invasive infections. Clin Infect Dis 2020; 71:e650–6. 32239136 10.1093/cid/ciaa365 PMC7745005

[54] Milgrom A . Use of dalbavancin in facilitating discharge of high risk patients in low resource settings. Open Forum Infect Dis 2020; 7(Suppl 1):S358.

[55] Moore N, Kohut M, Stoddard H, et al Health care professional perspectives on discharging hospitalized patients with injection drug use-associated infections. Ther Adv Infect Dis 2022; 9:20499361221126868. 36225855 10.1177/20499361221126868 PMC9549088

[56] Morrisette T, Miller MA, Montague BT, Barber GR, McQueen RB, Krsak M. Long-acting lipoglycopeptides: “lineless antibiotics” for serious infections in persons who use drugs. Open Forum Infect Dis 2019; 6:ofz274. 31281868 10.1093/ofid/ofz274 PMC6602887

[57] Nakagami P, Morita K, Schultz SK, Rodriguez L. Single center dalbavancin experience: a cost saving surprise in people with substance use disorder. Open Forum Infect Dis 2022; 9(Suppl 2):S666–7.

[58] O’Callaghan K, Tapp S, Hajkowicz K, Legg A, McCarthy KL. Outcomes of patients with a history of injecting drug use and receipt of outpatient antimicrobial therapy. Eur J Clin Microbiol Infect Dis 2019; 38:575–80. 30680563 10.1007/s10096-018-03461-3

[59] O’Rourke E, Maguire C, Torgersen J, Talati NJ, Binkley A. Impact of dalbavancin as step-down or salvage therapy on duration of hospitalization among people who inject drugs. Open Forum Infect Dis 2022; 9(suppl 2):S688.

[60] Papalekas E, Patel N, Neph A, Moreno D, Zervos M, Reyes KC. Outpatient parenteral antimicrobial therapy (OPAT) in intravenous drug users (IVDUs): epidemiology and outcomes. Open Forum Infect Dis 2014; 1(Suppl 1):S52–3.

[61] Pineo T, Goldman JD, Swartzentruber G, Kanderi T, Qurashi H, Dimech C. An observational study on the use of long acting buprenorphine (Sublocade) and a tamper resistant PICC for outpatient IV antibiotic administration in patients with serious infections and opioid use disorder; the STOP OUD project. Drug Alcohol Depend Rep 2022; 2:100020. 36845901 10.1016/j.dadr.2021.100020 PMC9948820

[62] Price CN, Solomon DA, Johnson JA, Montgomery MW, Martin B, Suzuki J. Feasibility and safety of outpatient parenteral antimicrobial therapy in conjunction with addiction treatment for people who inject drugs. J Infect Dis 2020; 222(Suppl 5):S494–8. 32877541 10.1093/infdis/jiaa025 PMC7566637

[63] Rizvi H, Baratz N, Hadid H, et al Outpatient parenteral antimicrobial therapy (OPAT) in injection drug users (IDUs): is it safe? Open Forum Infect Dis 2018; 5(Suppl 1):S306.

[64] Roberts K, Jordan R, Pearson B. In hospital addiction treatment reduces readmissions for patients with related IE [poster abstract]. J Addict Med 2022; 16:e328–e329.

[65] Rolfe RJ Jr, Mathews RE, Rodriguez JM, et al Implementation of a standardized protocol for hospitalized patients who inject drugs and require long-term antibiotics reduces length of stay without increasing 30-day readmissions. Open Forum Infect Dis 2017; 4(Suppl 1):S340–1.

[66] Ruiz-Conejo M, El-Dalati S, Stoner BJ. Alternatives to guideline directed therapy in bacterial endocarditis. Open Forum Infect Dis 2022; 9(Suppl 2):S787.

[67] Russo TA, Ritchie HR, Schimmel JJ, Lorenzo MP. Dalbavancin use in persons who use drugs may increase adherence without increasing cost. J Pharm Technol 2024; 40:3–9. 38318254 10.1177/87551225231205738 PMC10838542

[68] Shihadeh KC, Young H, Wyles DL, Jenkins TC. Evaluation of standardized dalbavancin use to facilitate early hospital discharge for patients inappropriate for outpatient parenteral antibiotic therapy. Open Forum Infect Dis 2019; 6(suppl 2):S336–7. 10.1093/ofid/ofaa293 PMC7415304 32793767

[69] Sikka MK, Gore S, Vega T, Strnad L, Gregg J, Englander H. “OPTIONS-DC,” a feasible discharge planning conference to expand infection treatment options for people with substance use disorder. BMC Infect Dis 2021; 21:772. 34372776 10.1186/s12879-021-06514-9 PMC8351414

[70] Solomon DA, Beieler AM, Levy S, et al Perspectives on the use of outpatient parenteral antibiotic therapy for people who inject drugs: results from an online survey of infectious diseases clinicians. Open Forum Infect Dis 2023; 10:ofad372. 37520410 10.1093/ofid/ofad372 PMC10372854

[71] Solomon DA, Price C, Johnson JAA, Montgomery MW, Martin B, Suzuki J. Can integration of addiction treatment facilitate safe discharge on OPAT for patients with infectious complications of injection drug use? Open Forum Infect Dis 2019; 6(Suppl 2):S341–2.

[72] Stockwell E, Rinehart K, Boes E, et al Outcomes of orthopaedic infections in recreational intravenous drug users requiring long-term antibiotic treatment. J Am Acad Orthop Surg Glob Res Rev 2022; 6:e22.00108. 10.5435/JAAOSGlobal-D-22-00108 PMC9191380 35696313

[73] Tan C, Shojaei E, Wiener J, Shah M, Koivu S, Silverman M. Risk of new bloodstream infections and mortality among people who inject drugs with infective endocarditis. JAMA Netw Open 2020; 3:e2012974. 32785635 10.1001/jamanetworkopen.2020.12974 PMC7424403

[74] Terriff C . Transition of care with dalbavancin: a successful cost-saving stewardship program through decreased length of stay. Open Forum Infect Dis 2017; 4(Suppl 1):S491.

[75] Traver EC, Ching PR, Narayanan S. Medication for opioid use disorder at hospital discharge is not associated with intravenous antibiotic completion in post-acute care facilities. Ther Adv Infect Dis 2022; 9:20499361221103877. 35755123 10.1177/20499361221103877 PMC9218897

[76] Van Hise NW, Anderson M, McKinsey D, et al The use of dalbavancin for *Staphylococcus aureus* bacteremia in persons who inject drugs (PWID). Open Forum Infect Dis 2019; 6(Suppl 2):S772.

[77] Vazirian M, Jerry JM, Shrestha NK, Gordon SM. Outcomes of outpatient parenteral antimicrobial therapy in patients with injection drug use. Psychosomatics 2018; 59:490–5. 29685397 10.1016/j.psym.2018.02.005

[78] Wildenthal JA, Atkinson A, Lewis S, et al Outcomes of partial oral antibiotic treatment for complicated *Staphylococcus aureus* bacteremia in people who inject drugs. Clin Infect Dis 2023; 76:487–96. 36052413 10.1093/cid/ciac714 PMC10169408

[79] Yang WT, Dombrowski JC, Glick SN, et al Partial-Oral antibiotic therapy for bone and joint infections in people with recent injection drug use. Open Forum Infect Dis 2023; 10:ofad005. 36726538 10.1093/ofid/ofad005 PMC9887258

[80] Zhou Y, Beieler A, Dhanireddy S. Outpatient antibiotic treatment outcomes in vulnerable populations: homeless and current injection drug users. Open Forum Infect Dis 2016; 3(Suppl 1):1330.

[81] Hurley H, Sikka M, Jenkins T, Cari EV, Thornton A. Outpatient antimicrobial treatment for people who inject drugs. Infect Dis Clin North Am 2020; 34:525–38. 32782100 10.1016/j.idc.2020.06.009

[82] Degenhardt L, Peacock A, Colledge S, et al Global prevalence of injecting drug use and sociodemographic characteristics and prevalence of HIV, HBV, and HCV in people who inject drugs: a multistage systematic review. Lancet Glob Health 2017; 5:e1192–207. 29074409 10.1016/S2214-109X(17)30375-3 PMC5683738

