# Supplementary material for: Management of People Who Inject Drugs With Serious Injection-Related Infections in an Outpatient Setting: A Scoping Review
Source: Open Forum Infect Dis. 2024 Oct 10;11(11):ofae613. doi: 10.1093/ofid/ofae613 (PMC11530960; doi:10.1093/ofid/ofae613)
Supplement: ofae613_Supplementary_Data [file ofae613_supplementary_data.docx]

Supplement Table 1. Embase search strategy

| **Injection drug user/** | **Bacterial infection/** | **Outpatient/** |
| --- | --- | --- |
| 1. **Intravenous drug abuse/** 2. Inject* drug use*.mp 3. Inject* substance use*.mp 4. Inject* opioid use*.mp 5. Inject* adj3 abuse*.mp 6. Intra* drug* use*.mp 7. Intra* substance use*.mp 8. Intra* opioid use*.mp 9. Intra* adj3 abuse*.mp 10. PWID.mp 11. IVDU.mp 12. RIVDU.mp 13. IVD.mp 14. PWUD.mp 15. People adj3 drug*.mp 16. Person* adj3 drug*.mp 17. People adj3 substance*.mp 18. Person* adj3 substance*.mp 19. People adj3 opioid*.mp   Person* adj3 opioid*.mp | 1. **Bacteremia/** 2. Bacteremia.mp 3. Bacteraemia.mp 4. **Bloodstream infection/** 5. Infection*.mp 6. **Bacterial endocarditis/** 7. Endocarditis.mp 8. **Central nervous system bacterial infection/** 9. Bacterial CNS infection*.mp 10. Bacterial infection* of the CNS.mp 11. Bacterial infections of the central nervous system.mp 12. Discitis.mp 13. **Abscess/** 14. Abscess.mp 15. **Osteomyelitis/** 16. Osteomyelitis.mp 17. **Infective endocarditis/** 18. **Empyema/** 19. Empyema.mp 20. **Prosthesis infection/** 21. **Infectious arthritis/** 22. Prosthetic adj2 infection*.mp 23. Hardware infection*.mp 24. **Bacterial arthritis/** 25. Arthritis.mp 26. **Antimicrobial therapy/** 27. Antibiotic*.mp 28. **Antibiotic therapy/** 29. **Antibiotic agent/** | 1. **Outpatient care/** 2. Outpatient*.mp 3. Out-patient*.mp 4. OPAT.mp 5. Outpatient adj2 antibiotic therapy.mp 6. Skilled nursing facility.mp 7. Residential treatment.mp 8. Residential.mp 9. Dalbavancin.mp 10. Telavancin.mp 11. Oritavancin.mp 12. **Oral drug administration/** 13. Oral adj2 administration.mp 14. Administration, oral.mp 15. Drug administration, oral.mp 16. Oral administration.mp 17. Oral drug intake.mp 18. po administration.mp 19. po dos*.mp 20. po drug*.mp 21. per os drug*.mp 22. Po antibiotic*.mp 23. Oral antibiotic*.mp |

The terms within column are combined with “OR” and the 3 columns are combined with “AND”

Supplement Table 2. Medline and Cochrane search strategy

| **Substance abuse, intravenous/** | **Bacterial infections/** | **Outpatients/** |
| --- | --- | --- |
| 1. Inject* drug use*.mp 2. Inject* SUBSTANCE use*.mp 3. Inject* OPIOID use*.mp 4. Inject* adj3 abuse*.mp 5. Intra* drug* use*.mp 6. Intra* substance use*.mp 7. Intra* opioid use*.mp 8. Intra* adj3 abuse*.mp 9. PWID.mp 10. IVDU.mp 11. RIVDU.mp 12. IVD.mp 13. PWUD.mp 14. People adj3 drug*.mp 15. Person* adj3 drug*.mp 16. People adj3 substance*.mp 17. Person* adj3 substance*.mp 18. People adj3 opioid*.mp   Person* adj3 opioid*.mp | 1. **Bacteremia/** 2. Bacteremia.mp 3. Bacteraemia.mp 4. Blood stream infection.mp 5. Infection*.mp 6. **Endocarditis, bacterial/** 7. Endocarditis.mp 8. **Central nervous system bacterial infections/** 9. Bacterial CNS infection*.mp 10. Bacterial infection* of the CNS.mp 11. Bacterial infections of the central nervous system.mp 12. Discitis.mp 13. **Abscess/** 14. Abscess.mp 15. **Osteomyelitis/** 16. Osteomyelitis.mp 17. **Empyema/** 18. Empyema.mp 19. **Prosthesis-related infections/** 20. **Arthritis, infectious/** 21. Prosthetic adj2 infection*.mp 22. Hardware infection*.mp 23. Arthritis.mp 24. **Anti-bacterial agents/**   Antibiotic*.mp | 1. Outpatient*.mp 2. Out-patient*.mp 3. OPAT.mp 4. Outpatient adj2 5. **Skilled nursing facilities/** 6. Skilled nursing facility.mp 7. **Residential treatment/** 8. Residential treatment.mp 9. Residential.mp 10. Dalbavancin.mp 11. Telavancin.mp 12. Oritavancin.mp 13. **Administration, oral/** 14. Po antibiotic.mp 15. Administration, oral.mp 16. Drug administration, oral.mp 17. Oral administration.mp 18. Oral drug intake.mp 19. po administration.mp 20. po dos*.mp 21. po drug*.mp 22. per os drug*.mp 23. Po antibiotic*.mp   Oral antibiotic*.mp |

The terms within column are combined with “OR” and the 3 columns are combined with “AND”

Supplement Table 3. CINAHL search strategy

| People who inject dugs | Bacterial infection | Outpatient care |
| --- | --- | --- |
| People who inject drugs or PWID  Injection drug users or persons who inject drugs  Injection drug use  Intravenous substance abuse  Intravenous drug users or injection drug users or IVDU or IV drug users  Intravenous drug users  Intravenous drug use  Persons who inject drugs  Persons who inject drugs or person who injects drugs or PWID or people who inject drugs | Bacteremia or bacteraemia or blood stream infection  Bacteremia or catheter-related infections  Infective endocarditis  Central nervous system infections  Discitis  Abscess  Osteomyelitis  Empyema  Prosthetic joint infection  Septic arthritis  Infectious arthritis  Antibiotics | Outpatient clinics or ambulatory care or outpatient services or outpatient care  OPAT or outpatient antibiotic therapy  Skilled nursing facility  Residential treatment or residential placement or residential intervention or residential care  Dalbavancin.mp  Telavancin.mp  Oritavancin.mp  Oral antibiotics  Oral antibiotic therapy  Oral drug delivery  Oral drug |

The terms within column are combined with “OR” and the 3 columns are combined with “AND”

Supplement Table 4. Description of included studies

| Author | Design | Study period | Country | Theme | PICO | Main results/Conclusions |
| --- | --- | --- | --- | --- | --- | --- |
| Adams | Decision analytical modeling study | 2020 | United States | Comparison of inpatient vs. outpatient management | P: 5 million patients with injection drug use-associated infecitve endocarditis (simulated)  I: 4-6 weeks IV antibiotics with inpatient addiction care services with OAT (usual care/ addictions care strategy)  3 weeks inpatient IV antibiotics with addictions care services followed by OPAT (OPAT)  3 weeks IV antibiotics with addictions care services, followed by partial oral antibiotics (partial oral)  C: 4-6 weeks inpatient IV antibiotics with opioid detoxification (usual care)  O: Life expectancy, mean cost per person, incremental cost-effectiveness ratios | Decision analytical modeling study simulating 5 million individuals with injection drug use-associated infective endocarditis which showed that outpatient IV antibiotic therapy and partial oral antibiotic therapy regimens were likely as clinical beneficial and less costly than 6 weeks of IV antibiotic therapy. |
| Agrawal | Quasi-experimental (before and after) study | 2022 | United States | Special settings for outpatient antibiotic delivery | P: Patients with IVDU-related infections  I: Multidisciplinary interventions addressing addictions (*n* = 64 admissions)  C: No multidisciplinary interventions (*n* = 80 admissions)  O: Rate of AMA discharges, rate of IV antibiotics on discharge, length of hospital stay, readmission rate | Involvement of a multidisciplinary team in patients with IVDU-related infections resulted in more patients discharged on outpatient IV antibiotics, overall lower length of stay, and more addictions issues being addressed, but resulted in similar rates of AMA and higher rates of readmissions. |
| Ahiskali | Retrospective case series | 2017 to 2019 | United States | OPAT vs. long-acting glycopeptide vs. partial oral | P: Adult PWID who received at least one dose of oritavancin for a gram positive infection  I: At least one dose of oritavancin  C: None  O: Clinical cure | Use of at least one dose of oritavancin for a gram-positive infection in PWID resulted in clinical cure in 19/24 cases. |
| Ajaka | Retrospective case series | 2017 to 2019 | United States | OPAT vs. long-acting glycopeptide vs. partial oral | P: Adult patients who received at least one dose of dalbavancin for bacteremia or IE  I: At least one dose of dalbavancin  C: None  O: Clinical or biologic cure | Use of at least one dose of dalbavancin for bacteremia or IE in PWID results in clinical cure in 8/18 cases. |
| Alosaimy | Retrospective cohort study | 2017 to 2019 | United States | OPAT vs. long-acting glycopeptide vs. partial oral | P: Adult patients who received at least one does of dalbavancin for any indication (predominantly PWID) including bacteremia, bone/ joint infections, and ABSSSI  I: At least one dose of dalbavancin  C: None  O: Clinical success (30-day survival, resolution of signs and symptoms of infection, absence of therapy escalation/ change) | Use of at least one dose of dalbavancin for any indication results in clinical success in 24/30 cases. |
| AlSalman | Retrospective case series | 2015 to 2019 | United States | Special settings for outpatient antibiotic delivery | P: Patients planned to receive at least one dose of Dalbavancin in a rural medical center (predominantly PWID), *n* = 33  I: At least one dose of dalbavancin  C: None  O: Completion of planned duration of therapy | In those planned to receive dalbavancin in a rural medical center, 15/33 cases were not completed, of which all were PWID, and 11/13 were lost to follow-up. Dalbavancin in rural centers may have high rates of non-compliance and many were lost to follow-up. |
| Ashraf | Retrospective case series | 2017 to 2018 | United States | Special settings for outpatient antibiotic delivery | P: PWUD requiring extended intravenous antibiotics for infections such as osteomyelitis, *n* = 83  I: Discharge to skilled nursing facility  C: None  O: Completion of antibiotics, AMA, 30 day readmission rates, ED utilization | In PWUD who are discharged to skilled nursing facilities for prolonged intravenous antibiotics, 26/83 left AMA and 20/83 early non AMA. Those who left AMA or early non-AMA had increased rates of 30-day admissions or ED utilization, or increased rates of 30-day readmissions. |
| Baddour | Scientific statement | 2021 | United States | Adjunctive addiction management | n/a | Writing group consisted of nine recognized experts in infectious diseases, cardiology, addictions medicine, and cardiovascular surgery conducted a literature search included 1345 articles to address diagnosis, management, and prevention of infective endocarditis among people who inject drugs. Highlights from statement include importance for concurrent treatment of substance use disorder with opioid agonist therapy, interventions such as harm reduction for injection drug use, importance of a multidisciplinary team, determining patients who may be appropriate for OPAT, options for oral antibiotics in Staphylococcus infective endocarditis, and timing, types, and concerns with regards to surgical interventions. |
| Beieler | Retrospective case series | 2015 to 2016 | United States | OPAT vs. long-acting glycopeptide vs. partial oral | P: Patients enrolled in OPAT who required >2 weeks of IV therapy including homeless PWID, housed PWID, homeless non-PWID, total *n* = 596  I: OPAT  C: None  O: Clinical cure (completion of antibiotics and resolution of infection), length of stay, secondary bacteremia, line-tampering, 30-day readmissions | In an OPAT, assuming patients lost to follow-up failed therapy, homeless PWID were least likely to achieve cure followed by housed PWID, followed by housed non-PWID. Cure rates did not differ in those not lost to follow-up. |
| Beieler | Retrospective case series | 2012 to 2014 | United States | Special settings for outpatient antibiotic delivery | P: Homeless patients >18 years old (predominantly current or remote injection drug use) who required IV antibiotics post discharge, *n* = 53  I: Antibiotics via OPAT at medical respite with a multidisciplinary team  C: None  O: Successful completion of antimicrobial course | In 53 homeless patients (predominantly injection drug users) who were received IV antibiotics via OPAT at medical respite, 46 successfully completed a defined course of antibiotics, 34 were successfully treated, 3% were readmitted, and average length of OPAT stay was 22 days. Cost savings to this institution was $25,00 per episode of OPAT. |
| Bird | Retrospective case series | 2017 to 2018 | United States | Special settings for outpatient antibiotic delivery | P: Patients with substance use disorder requiring prolonged antibiotics (predominantly injection drug use), *n*  = 76  I: Skilled nursing facility for OPAT  C: None  O: Completion of antibiotic course, hospital readmission | 56 out of 76 patients with substance use (predominantly injection drug use) who were discharged to skilled nursing facility for completion of IV antibiotics completed their antibiotic course. Of these patients, those who had history of injection drug use had a greater proportion of incomplete OPAT treatment, left AMA, and 30-day readmission rates, compared to those who did not have history of injection drug use. |
| Bork | Retrospective case series | 2014 to 2017 | United States | OPAT vs. long-acting glycopeptide vs. partial oral | P: Adults who received at least one dose of dalbavancin (predominantly PWID) for a non-ABSSSI indication, *n* = 28  I: At least one dose of dalbavancin  C: None  O: 30 and 90-day clinical cure and adverse drug events | In adults (predominantly PWID) who received at least one dose of dalbavancin for indications including osteomyelitis, endovascular infections, bacteremias, and ABSSSI, clinical cure was achieved in 15/21 evaluable patients. The remainder were lost to follow-up. |
| Bryson-Cahn | Retrospective case series | 2015 to 2017 | United States | OPAT vs. long-acting glycopeptide vs. partial oral | P: PWID who received dalbavancin for serious Staphylococcal infections (endocarditis, osteomyelitis, septic thrombophlebitis, epidural infections), *n* = 32  I: Dalbavancin  C: None  O: Clinical response | In PWID who received dalbavancin for serious Staphylococcal infections, the majority of patients (18/32) had clinical cure, and only 1 had treatment failure. |
| Buehrle | Retrospective case series | 2013 to 2015 | United States | Comparison of inpatient vs. outpatient management  Predictive factors | P: IVDUs receiving IV antibiotics via OPAT, *n*  = 67  I: IV antibiotics via OPAT  C: None  O: OPAT failure, defined as worsening or ongoing infection requiring hospital readmission, worsening or ongoing infection resulting in prolonged antibiotics, antibiotic noncomplaince, noncompliance with follow-up clinic appointments, death during traetment course | Among IVDUs receiving IV antibiotics via OPAT, there was OPAT failure in 61% (41/67) of patients. Reasons for failure include 30-day readmission, worsening infection requiring prolonged antibiotics, missed clinic follow-up visits, noncompliance with antibiotic therapy, death, and documented line manipulation. Risk factors for OPAT failure included more recent IVDU. |
| Camsari | Retrospective case series | 2014 to 2016 | United States | Special settings for outpatient antibiotic delivery | P: Patients with addictions requiring prolonged IV antibiotics with PICC in US small towns  I: Psychosocial risk factor stratification   1. High risk, *n* = 10 2. Moderate risk, *n* = 5 3. Low risk, *n* = 5   C: None  O: Compliance with antimicrobial therapy, illicit drug use during antibiotic course | In patients with addictions disorders requiring prolonged IV antibiotics in US small towns, use of a psychosocial risk factor stratification model to stratify patients to high, moderate, and low risk can be used to determine which patients will successfully comply with antimicrobial therapy and have illicit drug use during antibiotic course. Those considered moderate and low risk can be safely discharged for OPAT with a PICC. |
| Narayan | Retrospective cohort | Not specified | United States | Predictive factors | P: Patients with substance use disorders with infections requiring prolonged antibiotics for infections including osteoarticular infections, infective endocarditis, endovascular infections, *n* = 263  I: Antibiotic courses  C: No  O: Management, SUD interventions, and parenteral outcomes between groups | An analysis of characteristics of patients with substance use disorder who received antibiotic courses. 79% of antibiotic courses were completed in skilled nursing facilities, 64% had consultation by substance use services, 68% had documentation of medications for opioid use disorder at discharge. Completion of therapy was documented in 62% of encounters. Non-adherence was documented in 32% of episodes with documented injection drug use, 28% with active substance use in prior year, and 33% of encounters where use of more than one substance was used. Drug or catheter related related averse events were higher in IDu group and catheter abuse documented in 7 encounters of which 6 were with IDU or active SUD documented. An unfavorable outcome occurred in 58% of IDU encounters compared to 42.5% of non-IDU encounters. |
| D’Couto | Retrospective cohort | 2010 to 2015 | United States | Special settings for outpatient antibiotic delivery | P: PWID with injection drug use in preceding two years who required at least 2 weeks of IV antibiotics via OPAT  I:  IV antibiotics via OPAT at home  C: IV antibiotics via OPAT at skilled nursing facility  O: Completion of antibiotic courses, line infections, injection drug relapse, readmission | For PWID who require antibiotics via OPAT, those who were discharged home were not more likely to have complications than those discharged to skilled nursing facilities. |
| Dobson | Retrospective cohort study | 1995 to 2017 | Australia | Comparison of inpatient vs. outpatient management  OPAT vs. long-acting glycopeptide vs. partial oral | P: IDU receiving OPAT at home for bone and joint infections, endocarditis, bacteremia, abscess, SSTIs, and others, *n* = 159  I: OPAT in home environment  C: Non IDU receiving OPAT at home for bone and joint infections, endocarditis, bacteremia, abscess, SSTIs, and others, *n* = 6493  O: Compliance with OPAT, early discharge from OPAT program due to a complication, readmissions to hospital, use of after-hours on-call nurse to troubleshoot a catheter complication, catheter related thrombosis or catheter-related bloodstream infections, catheter blockage and damage, accidental catheter removal/ dislodged catheters | When compared to non IDU using OPAT, IDU using OPAT were more likely to be noncompliant during OPAT, less likely to be discharged from OPAT due to a complication, more likely to contact the after hours on-call nurse for a telephone consultation, more likely to have line failure, and more likely to have accidental catheter removal/ dislodged catheter. There was no difference between both groups for removal of catheter for any complication. |
| Douglass | Retrospective case series | 2018 to 2022 | United States | Multidisciplinary discharge planning | P: PWUD who require >2 weeks of antibiotics for infections including bacteremia, endocarditis, and osteomyelitis, *n* = 144 conferences  I: OPTIONS-DC (structured multidisciplinary care conference)  C: None  O: Initiation of medication assisted therapy for OUD, antibiotic completion rates | Use of a structured multidisciplinary care conference that incorporates harm reduction principles and patient preferences for PWUD on discharge for those who require >2 weeks of antibiotics results in favorable results in terms of completion of antibiotic course (112/144), initiation of medication assisted therapy (79 of 106 with opioid use disorder). Antibiotic course completion rates were similar for those who were discharged home or to a skilled nursing facility. With the OPTIONS-DC conference, the patient directed discharge rate is 8.3%, which is lower than what is generally reported in this scenario. |
| Douglass | Retrospective case series | 2015 to 2019 | United States | OPAT vs. long-acting glycopeptide vs. partial oral | P: Adult patients with documented substance use (predominantly intravenous) who received at least one does of dalbavancin, *n* = 53 courses, for indications including bone and joint infections, endocarditis, and SSTI  I: At least one dose of dalbavancin  C: None  O: Number of interventions required by OPAT RNs and other OPAT staff for coordination of dalbavancin | In an OPAT providing dalbavancin to substance users, RN intervention was required in coordination of 60% of courses and 77% of courses in which at least one outpatient dose was needed. 8.9 minutes were needed in RN intervention per dalbavancin course. |
| Eaton | Retrospective case series | 2015 to 2016 | United States | Multidisciplinary discharge planning | P: PWID who require intravenous antibiotics  I: 9-point risk assessment to identify patients who were safe for discharge  C: None  O: Length of stay, total direct cost | Use of a 9-point assessment to determine low-risk patients who can be safely discharged with outpatient antibiotics reduces mean length of stay by 20-days, and total direct cost by 30%. |
| Eckland | Qualitative interviews | 2021 to 2022 | United States | Perceptions | n/a | Qualitative interviews were held with patients (*n* = 10) hospitalized with IDU-associated infections and with community partners (*n* = 6). Interviews revealed that patients with IDU-associated infections desire autonomy, respect, and patient centered care from healthcare workers, and may self-discharge when preferences or needs are not met. |
| Englander | Mixed methods study | 2016 | United States | Perceptions | P: Patients with substance use disorder requiring prolonged  IV antibiotics (>2 weeks)  I: Medically enhanced residential treatment (MERT), *n* = 7  C: None  O: Completion of antibiotic course | Of 45 patients with substance use disorder requiring prolonged antibiotics, 18 were ineligible and 20 declined MERT, and of the 7 who enrolled in MERT, only 3 completed antibiotic IV antibiotics suggesting that there remains many challenges to its implementation. Interviews revealed that barriers include patient ambivalence to residential treatment, wanting to prioritize physical health needs, and fears of untreated pain in the residential treatment center. |
| Fanucchi | Randomized controlled trial (ongoing) | 2021 to 2022 | United States | Comparison of inpatient vs. outpatient management | P: Adults hospitalized with OUD and severe injection related infections requiring >2 weeks of IV antibiotics, *n* = 90  I: Discharge once medically stable to integrated outpatient care model combining Buprenorphine and OPAT  C: Treatment as usual  O: Length of stay, length of OPAT, serious adverse events including death, hospitalization, overdose | Patients with opioid use disorder and severe injection related infections were randomized to treatment as usual, or to an integrated outpatient care model combining buprenorphine and OPAT. Preliminary results suggest that there are very severe adverse events in the integrated outpatient care model (including overdose deaths, hospitalizations etc). |
| Fanucchi | Qualitative survey by email | 2015 | United States | Perceptions | n/a | Semistructured interviews were conducted with hospital physicians, surgeons, cardiologists, and infectious diseases physicians to attitudes towards IDU in OPAT. 95% use OPAT in those without IDU, but only 29% would consider OPAT in PWID. 79% would consider OPAT for those with remove IDU. There was no agreed upon definition on remote history of IDU. Common physician identified barriers to discharging PWID on OPAT include socioeconomic factors, risk of misusing PICC for IDU, and willingness of an ID physician to follow these patients as outpatients. |
| Fanucchi | Randomized pilot study | 2018 | United States | Adjunctive addiction management | P: Persons with opioid use disorder hospitalized with injection-related infections requiring >2 weeks of IV antibiotics  I: Care model combining outpatient parenteral antimicrobial therapy with buprenorphine treatment, *n* = 10  C: Usual care (completing IV antibiotics in hospital), *n* = 10  O: Clinical and drug use outcomes, length of stay | In a randomized study, patients with opioid use disorder who have serious injection related infections who require prolonged antibiotics, there were similar clinical outcomes between those who received usual care (completing IV antibiotics in hospital) compared to those who received antibiotics at an OPAT with a care model combining buprenorphine treatment with IV antibiotics. Length of stay on average was shortened by 23.5 days. |
| Fanucchi | Case series | 2017 to 2018 | United States | Adjunctive addiction management | P: Patients with opioid use disorder requiring OPAT for prolonged antibiotics, *n* = 3  I: Comprehensive outpatient model incorporating OUD treatment with buprenorphine and counseling with OPAT  C: None  O: Clinical success, length of stay | An illustration of three cases of patients with OUD who require OPAT for prolonged antibiotics, with a comprehensive outpatient model currently executed in Kentucky that incorporates OUD treatment with buprenorphine counseling with OPAT. Successes and challenges with these cases were described, and the clinical activities and responsibilities of the patient within this model were discussed. |
| Gelman | Quasi-experimental study pre-/post-study design | 2017 to 2018 | United States | Adjunctive addiction management | P:  PWID with serious bacterial infections  I: Comprehensive Care of Drug Addiction and Infection (CCDAI) program, which collaborates between the hospital system and a detoxification facility to provide simultaneous drug recovery assistance and parenteral antibiotic therapy, *n* = 35  C: Pre-implementation control group, *n* = 51  O: Completion of full outpatient parenteral antibiotic therapy, length of stay, cost, readmission rates, all-cause mortality | Partnership between hospitals and detoxification facilities for PWID with serious bacterial infections result in significant reductions in length of stay and cost without increases in readmission rates, and possibly decrease in 1 year mortality rates. |
| Greco | Retrospective cohort study (quasi-experimental pre- and post- design) | 2017 to 2019 | United States | Adjunctive addiction management | P: People with opioid use disorder with Staphylococcus aureus bacteremia receiving treatment with OPAT  I: Medication assisted treatment for substance use disorder during OPAT, *n* = 17  C: No medical assisted treatment during OPAT, *n* = 27  O: Composite outcome of failure to complete OPAT, recurrence of Staphylococcus aureus bacteremia during OPAT period, readmission within 30 days, length of stay | For people with opioid use disorder who received antibiotics via OPAT for Staphylococcus aureus bacteremia, those who received medical assisted treatment for opioid use disorder had shorter length of stay, and were more likely to meet the primary composite outcome of not completing OPAT, recurrence of Staphylococcus aureus bacteremia, or readmission within 30 days. |
| Heil | Retrospective case series | 2016 to 2018 | United States | OPAT vs. long-acting glycopeptide vs. partial oral | P: Patients who received dalbavancin in an ED for SSTI (predominantly PWID), *n* = 24  I: Dalbavancin  C: None  O: 7-day ED revisit after dalbavancin, hospital admission, 7-day ED revisit for non-SSTI indication, outpatient follow-up attendance | In those who received dalbavancin in ED for SSTI, 7/24 patients returned to ED within 7-days with chief complaint of SSTI, 7/24 attended scheduled 14-day outpatient follow-up visit, 2/24 were admitted from ED and 4/24 had an ED visit within 14-days for a non-SSTI indication. |
| Ho | Cross sectional survey | 2010 | International (Australia, New Zealand, Asia, North America, UK, Europe) | Perceptions | n/a | Surveys were conducted to OPAT centers over the world which identified that 84% of centers treat patients with history of IVDU, and a significant proportion use peripherally inserted central catheters. Most responders believe that use of OPAT in IVDU is beneficial and outweighs the risk with little interregional variation. |
| Ho | Prospective case series | 2005 to 2009 | Singapore | OPAT vs. long-acting glycopeptide vs. partial oral | P: IVDU patients requiring parenteral antibiotics via an OPAT, *n* = 29  I: Parenteral antibiotics via an OPAT  C: None  O: Mortality, completion of therapy, PICC abuse, readmission for infective or treatment-related complications during OPAT, 30-day follow-up period | In 29 IVDU patients who received IV antibiotics via an OPAT, 5 patients during OPAT and 1 patient during the 30-day follow-up period required readmission for infective or treatment-related complications, and 2 had recrudescent infection after being lost to follow-up which were subsequently successfully treated in OPAT. |
| Hoff | Case series | 2021 | United States | Special settings for outpatient antibiotic delivery | P: Patients with addiction with infections requiring prolonged IV antibiotics, *n* = 10  I: Self administered outpatient parenteral antibiotic therapy  C: None  O: Completion of antibiotic therapy, follow-up appointment attendance, 30-day hospital readmissions | Patients with substance use disorder who require antibiotics for infections can successfully complete self administered outpatient parenteral antibiotic therapy. |
| Jafari | Retrospective mixed methods study | 2005 to 2009 | Canada | Multidisciplinary discharge planning  Special settings for outpatient antibiotic delivery | P: Patients requiring prolonged antibiotics for infections such as osteomyelitis, of which 39% were injection drug users, *n* = 165  I: Model to provide IV antibiotics to injection drug users in a community care setting  C: Treatment with IV antibiotics in hospital  O: Length of stay, AMA, discharge to stable housing, satisfaction | Use of a community care setting to deliver IV antibiotics to patients requiring prolonged antibiotics (many injection drug users) resulted in longer length of stay, lower rates of AMA, higher rates of satisfaction, and higher rates of discharge to stable housing. |
| Jewell | Retrospective case series | 2006 to 2011 | United States | Special settings for outpatient antibiotic delivery | P: Injection drug users with infections requiring IV antibiotics such as osteomyelitis or septic arthritis  I: Treatment with IV antibiotics at an addiction treatment facility  C: None  O: Antibiotic completion rates, illicit drug relapse, cost savings | Use of community residential addiction treatment facility for provide IV antibiotics for PWID results in a 73% completion rate of antibiotic courses, with a low relapse rate to illicit drug use of 32%, and results in cost savings of $2.43 million in a 6-year period. |
| Juskowich | Retrospective case series | 2020 to 2022 | United States | Multidisciplinary discharge planning  Special settings for outpatient antibiotic delivery | P: Patients requiring prolonged antibiotics typically requiring intravenous treatment (predominantly PWID) for infections including endocarditis and bone and joint infections, *n* = 100  I: Complex outpatient antimicrobial therapy using oral antibiotics with once/ week follow-up via phone  C: None  O: Number of IV antibiotic days, length of stay, hospital costs, 30-day readmission rate, follow-up rates | In a sample of 100 patients (predominantly PWID) who required prolonged IV antibiotics, transition to a complex outpatient antimicrobial therapy program providing oral antibiotics safely decreases hospital length of stay, and results in reduced hospital courses, with low 30-day readmission rates. |
| Kershaw | Quality improvement health care redesign study | 2019 to 2020 | United States | Multidisciplinary discharge planning | n/a | A multidisciplinary group of clinicians and patients used design-thinking-based redesign process to create an intervention with early identification of hospitalized patients who inject drugs with serious infections, proactive psychiatry consultation service for addiction management for all patients, multidisciplinary care conference to support decision making around treatment options for infection and substance use, and care coordination/ navigation in outpatient setting with substance use peer recovery coach and infectious disease nurse for patients discharged home on IV antibiotics. |
| Lewis | Prospective cohort study | 2020 to 2021 | United States | Multidisciplinary discharge planning  OPAT vs. long-acting glycopeptide vs. partial oral | P: PWID with serious injection-related infections  I: Oral antibiotics after patient directed discharge to complete antibiotic course, *n* = 105  C: IV antibiotics as an in-patient, *n* = 61  O: 90-day readmissions, substance use disorder clinic follow-up | No difference in 90-day readmission rates between PWID who completed IV antibiotics as an inpatient, and PWID who completed oral antibiotics after patient directed discharge. Factors protective against readmission after patient directed discharge include antibiotic and medications for OUD adherence, engagement with support team, and clinic follow-up. |
| Lueking | Retrospective case series | 2019 to 2021 | United States | OPAT vs. long-acting glycopeptide vs. partial oral | P: All patients that received at least one dose of dalbavancin in inpatient or outpatient setting (predominantly IDU), *n* = 40 for indications including endocarditis, septic arthritis  I: At least one dose of dalbavancin  C: None  O: Clinical failure, measured by avoidance of ED visits or hospital readmission at 30, 60, and 90-days | In those who received dalbavancin in an inpatient or outpatient setting (predominantly IDU and homeless), clinical failure was identified in only 5 of 40 patients. |
| Marks | Retrospective cohort study | 2016 to 2019 | United States | OPAT vs. long-acting glycopeptide vs. partial oral | P: PWID aged >17 with invasive infections requiring prolonged antibiotics  I:   1. Partial course of IV antibiotics not prescribed antibiotics on AMA discharge, *n* = 46 2. Partial course of IV antibiotics prescribed oral antibiotics on AMA discharge, *n* = 27   C: Full course of IV antibiotics, *n* = 43  O: 90-day readmission rates | In PWID who required antibiotics, 90-day readmission rates were highest amongst PWID who did not receive oral antibiotics on AMA discharge, followed by partial oral, followed by full inpatient antibiotics. Surgical source control and addictions medicine consultations were associated with reduced readmissions. |
| Milgrom | Retrospective case series | 2019 | United States | OPAT vs. long-acting glycopeptide vs. partial oral | P: PWID treated with dalbavancin to facilitate early discharge, *n* = 10  I: Dalbavancin  C: None  O: Length of stay, outpatient follow-up after discharge | Dalbavancin can help facilitate early discharge in PWID but follow-up for treatment remains abysmal making it difficult to assess for clinical response. |
| Moore | Qualitative interview study | 2022 | United States | Perceptions | n/a | Semistructured interviews with healthcare professionals (*n* = 19) with regards to outpatient treatment options for IDU associated infections. Healthcare practitioners may be unaware of existing community resources, and may believe that discharging patients into community exposes them to structural harms. Some HCPs are concerned that patients with substance use disorders will not make “good” decisions regarding outpatient antimicrobial options. Many have uncertainty about how responsibility for offering outpatient treatment is shared across changing care teams. |
| Morrisette | Retrospective case series | 2015 to 2018 | United States | OPAT vs. long-acting glycopeptide vs. partial oral | P: Adults treated with dalbavancin or oritavancin based on clinical judgment  I: Dalbavancin or oritavancin in PWUD, *n* = 17  C: Dalbavancin or oritavancin non PWUD, *n* = 39  O: Follow-up, clinical failure | Of adults who received treatment with dalbavancin or oritavancin, clinical failure and average length of stay was similar in those who use drugs and those who do not. Estimated median savings were $40,455 in PWUD and $19,555 in non PWUD. |
| Nakagami | Retrospective case series | 2019 to 2021 | United States | OPAT vs. long-acting glycopeptide vs. partial oral | P: Patients receiving dalbavancin (predominantly with substance use disorder), *n* = 34, for bone and joint infection, bacteremia  I: Dalbavancin  C: None  O: Adherence, representation for an infection, cost savings, adverse events | In those who received dalbavancin (predominantly in those with substance use disorders), 26/34 received their full prescribed antibiotic course, and 12/23 of those who were >90 days from a completed dalbavancin course represented for treatment of another infection. Only 1 adverse event was reported, and cost savings were calculated to be $1.47 million and 445 total hospital days were averted. |
| Norris | Guidelines | 2018 | United States | Comparison of inpatient vs. outpatient management | n/a | A panel of experts convened by IDSA updated the 2004 clinical practice guidelines for OPAT, providing recommendations with regards to patient considerations, antimicrobial utilization, vascular access devices, monitoring, and antimicrobial stewardship. |
| O’ Callaghan | Retrospective case series | 2015 to 2017 | Australia/ Germany | OPAT vs. long-acting glycopeptide vs. partial oral | P: PWID who underwent OPAT for prolonged antibiotic treatment for deep seated and endovascular infections, *n* = 38  I: OPAT  C: None  O: Successful completion of OPAT care with clinical improvement, hospital readmission, new bloodstream infections, patient non-compliance including ongoing non-prescribe IVDU, staff safety compromise | In PWID who received OPAT care for prolonged treatment of deep seated and endovascular infections, 28 or 38 episodes of OPAT care were completed successfully, with high rates of readmission, non-attendance, and line-associated infections. There were no adverse events for staff safety, and no patient deaths. |
| O’Rourke | Retrospective case series | 2019 to 2021 | United States | OPAT vs. long-acting glycopeptide vs. partial oral | P: PWID who received dalbavancin after completing intended IV antibiotic course (*n* = 11) and after patient directed discharge (*n* = 8)  I: One time dalbavancin dose  C: None  O: Readmissions, length of stay | In PWID who received dalbavancin after completing an intended IV antibiotic course and in those who left hospital via patient directed discharge, dalbavancin can help prevent readmissions and hospital length of stay. |
| Papalekas | Retrospective case series | 2011 to 2013 | United States | OPAT vs. long-acting glycopeptide vs. partial oral | P: IVDUs requiring OPAT, *n* = 39, for osteomyelitis, endocarditis, SSTI  I: OPAT either at home or group home (*n* = 10)  C: None  O: Deaths, readmissions, improvement/ cures relapse, lost to follow-up | In 39 IVDU patients who received OPAT care for prolonged antibiotics either at home or in a group home (of which there was information available for 30 patients), there were 0 deaths, 7 readmissions, 22 improvements/ cures, 1 relapse, and 9 loss to follow-up. |
| Pineo | Case series | 2020 to 2021 | United States | Adjunctive addiction management | P: Patients with opioid use disorder and serious infections, *n* = 13  I: Long-acting buprenorphine and tamper resistant clamp in PICCs for administration for IV antibiotics in an outpatient setting  C: None  O: Length of stay, cost savings | In 13 patients with opioid use disorder who concurrently required IV antibiotics via a PICC for intravenous antibiotics, 11 patients completed their antibiotic courses as prescribed, there was no evidence of PICC tampering, and institutional savings per patient was $33,000. Infections resolved in all patients. |
| Price | Retrospective case series | 2018 to 2019 | United States | Adjunctive addiction management | P: PWID requiring IV antibiotics for infections such as bacteremia, endocarditis, bone and joint infections, epidural abscess, *n* = 68  I: Addiction treatment combined with OPAT  C: None  O: Completion of antibiotics, 30-day readmission, relapse rate | In 20 out of 68 PWID who qualified for OPAT combined with addictions treatment, 100% completed antibiotic course, 30% experienced 30-day readmission, and 15% relapsed. There were no deaths ,overdoses, or PICC central-line complications. |
| Rizvi | Retrospective case series | 2011 to 2017 | United States | OPAT vs. long-acting glycopeptide vs. partial oral | P: Current or former IDU who received OPAT care, *n* = 61  I: OPAT care  C: None  O: Clinical cure (completed treatment and symptom free for 1 month after completion), improvement, relapsed (readmission within 30 days for same infection or sequelae), follow-up | Of 61 IDU who received OPAT care for antibiotics, 33 attended clinic follow-up, 18 demonstrated clinical cure, 14 demonstrated improvement, and 3 relapsed. |
| Roberts | Retrospective case series | 2020 to 2021 | United States | Adjunctive addiction management | P: PWID with IVDU related infections including osteomyelitis and infective endocarditis, *n* = 83  I: Multidisciplinary team with addiction medicine physicians, peer support services, harm reduction education, safe injection practices, resources for syringe service programs, connection to outpatient addiction treatment in inpatient and outpatient settings  C: None  O: Initiation of medications for opioid use disorder, and hospital readmissions at 30, 60, and 90 days | Of 83 PWID of IVDU related infections, 65 were discharged with medications for opioid use disorder, 4 with opioid agonists, 54% chose to follow the outpatient addiction treatment program. Patients who chose outpatient addiction treatment experienced >50% reduction of hospital readmission rates at 30 days, 60 days, and 90 days. |
| Rolfe | Quasi-experimental study before and after | 2017 | United States | Multidisciplinary discharge planning | P: PWID requiring prolonged IV antibiotics  I: Multidisciplinary team assessment including addictions medicine, infectious diseases to risk stratify patients into low, medium, and high risk, *n*  = 34  C: Pre-implementation group, *n* = 37  O: Rate of AMAs, rate of readmissions, length of stay | Use of a multidisciplinary team to risk stratify PWID for appropriateness for OPAT leads to improvement in LOS and addictions care for hospitalized PWID requiring long-term antibiotics. |
| Ruiz-Conejo | Retrospective case series | 2021 to 2022 | United States | OPAT vs. long-acting glycopeptide vs. partial oral | P: Patients requiring antibiotic treatment for endocarditis (predominantly with recent injection drug use)  I:   1. Partial oral antibiotics, *n* = 12 2. Dalbavancin, *n* = 5 3. Two week course of IV antibiotics after valve surgery   C: None  O: 90-day mortality, 30-day readmission rate | In those with predominant injection drug use, alternative methods to treat infective endocarditis including partial oral antibiotics, dalbavancin, or two week IV course after valve surgery may be feasible, with overall 90-day mortality rate of 0%, and 30-day readmission rate of 10%. 10/12 completed partial oral antibiotics as intended, ⅖ dalbavancin patients required transition to oral due to side effects. |
| Russo | Retrospective case series | 2020 to 2022 | United States | OPAT vs. long-acting glycopeptide vs. partial oral | P: PWUD who required treatment for Staphylococcus aureus bacteremia  I: Dalbavancin, *n* = 29  C: Standard care (ie. no dalbavancin), *n* = 20  O: Cost, treatment completion | Use of dalbavancin in PWUD for Staphylococcus aureus bacteremia may increase completion rates of intended treatment, but does not have statistically different cost overall. |
| Shihadeh | Retrospective case series | 2018 to 2019 | United States | OPAT vs. long-acting glycopeptide vs. partial oral | P: Patients requiring dalbavancin (predominantly injection drug users and homeless), *n* = 16 for gram-positive bacteremias, endocarditis, and osteomyelitis  I: One dose of dalbavancin 7-10 days prior to planned end of treatment  C: None  O: Safety and effectiveness 30-days after discharge | In patients requiring dalbavancin (predominantly injection drug users), there were no readmissions in all of 16 patients for indications related to infection or dalbavancin, 115 hospital days were averted, with cost savings to the hospital estimated to be $159,000. |
| Sikka | Retrospective case series | 2018 to 2019 | United States | Multidisciplinary discharge planning | P: Patients with substance use disorder (predominantly IV substance use) who require outpatient antibiotics, *n* = 55  I: OPTIONS-DC, a multidisciplinary interprofessional care conference  C: None  O: Completion of antibiotics, premature departure from hospital | Use of a multidisciplinary care conference for patients with substance use disorder (predominantly IV substance use) who require outpatient antibiotics leads to 70% completion of recommended antibiotic course, low (12%) rates of premature departure from hospital, allows recommendation of patient-centered antibiotic courses, exposes/ contextualizes SUB, psychosocial risk and protective factors, incorporates patient preferences, and allows providers to tailor antibiotic and SUD recommendations. |
| Solomon | Survey of ID clinicians | 2022 | United States | Perceptions | n/a | A voluntary survey was conducted of ID clinicians (*n* = 239) to determine practice patterns and attitudes regarding use of OPAT for PWID. 72% reported that PWID are eligible for OPAT, and 28% that they are not. Those who report that PWID are eligible to OPAT report more access to inpatient and outpatient social work/ case managers, more access to outpatient addictions services. There was no difference in access in inpatient addictions services, whether clinicians work in urban settings, or whether clinicians worked in urban settings. |
| Solomon | Retrospective cohort study | 2019 | United States | Adjunctive addiction management  Multidisciplinary discharge planning | P: Those receiving antibiotics via OPAT requiring prolonged antibiotics including for endocarditis, epidural abscess bone/ joint infections  I: PWID, *n* = 18  C: No history of IDU, *n* = 390  O: Relapse to substance use disorder, Line tampering, thrombosis, line infection, line dislodgement, deaths, overdoses | In 18 PWID who received prolonged antibiotics via an OPAT in which additions services were integrated, all individuals completed recommended courses of IV antibiotics, all OUD patients received OAT, two relapsed to drug use during OPAT but there were no instances of line tampering, thrombosis, line infection, line dislodgement. No deaths or overdoses were reported. There were no statistically significant difference in rates of readmission, line infections, mortality rate, ID clnic visit attendance, or number of days on OPAT between those who used and did not use drugs. |
| Stockwell | Retrospective cohort study | 2012 to 2018 | United States | Comparison of inpatient vs. outpatient management | P: Patients who orthopedics infections requiring long-term intravenous antibiotics (>4 weeks)  I: PWID, *n* = 123  C: Non PWID, *n* = 55  O: Treatment compliance, resolution of infection, catheter complications, loss to follow-up | Non PWID have more compliance to IV antibiotic courses than PWID for orthopedic infections. PWID who remained inpatients for antibiotic courses had better resolution of infection than those who did not. |
| Tan | Retrospective cohort study | 2007 to 2018 | Canada | Comparison of inpatient vs. outpatient management | P: PWID 18 years or older admitted with infective endocarditis, predominantly injection drug users, *n* = 420  I: Antibiotics in outpatient setting  C:  Antibiotics in inpatient setting  O: New BSI | New bloodstream infections complicate 20% of treatment courses for infective endocarditis in PWID, but it was not more common in those receiving outpatient treatment. |
| Terriff | Retrospective case series | 2015 to 2017 | United States | OPAT vs. long-acting glycopeptide vs. partial oral | P: Patients receiving dalbavancin (predominantly IVDUs) for complicated SSTI, osteomyelitis/ joint infections, and bacteremia  I: Dalbavancin  C: None  O: Readmissions, cost savings, adverse effects | Of 17 patients (predominantly IVDUs) who received dalbavancin for treatment of infections, only one patient was readmitted. Treatment was well tolerated with no complications, total length of stay was decreased by 270 days, and overall savings were $200,000. |
| Traver | Retrospective cohort study | 2017 to 2020 | United States | Adjunctive addiction management  Predictive factors | P: People with opioid use disorder (predominantly injection drug users) and severe infections such as osteoarticular infections discharged to a post-acute care facility  I: Opioid agonist therapy (methadone, buprenorphine) concurrently with with OPAT in post-acute care facilities, *n* = 65  C: No opioid agonist therapy with OPAT in post-acute care facilities, *n* = 33  O: Completion of outpatient parenteral antimicrobial therapy | For people with opioid use disorder (predominantly injection drug users) who were treated for infections in OPAT at post-acute care facilities, there was no statistically significant difference in completion of antibiotics in those who received opioid agonist therapy, and those who did not. |
| Van Hise | Retrospective case series | 2018 | United States | OPAT vs. long-acting glycopeptide vs. partial oral | P: PWID who have Staphylococcus aureus bacteremia, *n* = 16  I: 4-6 weekly doses of dalbavancin in outpatient setting  C: None  O: Readmissions in 6-months | Of 16 PWID who received weekly dalbavancin doses for Staphylococcus aureus bacteremia, only 2/6 were readmitted in 6-months for recurrent bacteremias related to injection drug use. 14/16 were disease free at 6-months. |
| Vazirian | Retrospective cohort study | 2013-2014 | United States | OPAT vs. long-acting glycopeptide vs. partial oral | P: Patients receiving antibiotics in OPAT  I: OPAT in IDU, *n* = 39  C: OPAT in those without IDU, *n* = 117  O: Treatment failure, infection relapse, line infection, hospital readmission, ED visits, 90-day mortality | There were no significant differences in clinical outcomes in IDU and non IDU treated in OPAT in terms of treatment failure, infection relapse, line infection, hospital readmissions, ED visits, and 90-day mortality. |
| Wildenthal | Retrospective cohort | 2016 to 2021 | United States | OPAT vs. long-acting glycopeptide vs. partial oral | P: Adult patients with history of injection drug use requiring antibiotics for Staphylococcal bloodstream infections, including infective endocarditis, epidural abscess, vertebral osteomyelitis, and septic arthritis  I:   1. Incomplete IV antibiotics 2. Transition from IV to partial oral   C: Standard of care (IV) antibiotics  O: Composite endpoint of death or readmission from microbiological failure within 90-days of discharge | In PWID who required antibiotics for Staphylococcal bloodstream infections, there was no statistically significant difference in microbiologic failure rates when comparing those who completed standard of care IV antibiotics versus partial oral antibiotics after receiving at least 10 days of IV antibiotics. |
| Yang | Retrospective cohort study | 2019 to 2021 | United States | OPAT vs. long-acting glycopeptide vs. partial oral | P: PWID who have bone and joint infections requiring >6 weeks of antibiotics  I: Partial oral antibiotics, *n* = 74  C: Exclusive IV antibiotics, *n* = 12  O: Rates of failure (defined as death, symptoms, or signs concerning for worsening or recurrent infections) 90 and 180 days after completion of antibiotics | In 74 PWID who received partial oral antibiotics for bone and joint infections, the failure rate was 20% at 90 days and 21% at 180 days. |
| Zhou | Retrospective case series | 2015 to 2016 | United States | OPAT vs. long-acting glycopeptide vs. partial oral | P: Patients receiving antibiotics via OPAT (many IDU), *n* = 688  I: Antibiotics via OPAT  C: None  O: Readmission rates, length of stay. | In 688 OPAT episodes, readmission rates were higher among current IDU (28%), than in overall OPAT population (20%), and length of stay was 2.1 days longer and readmission rates were 2 times higher in the homeless and current IDU. |
